# Evolutionary History of Bacteriophages in the Genus *Paraburkholderia*

**DOI:** 10.3389/fmicb.2018.00835

**Published:** 2018-05-11

**Authors:** Akbar Adjie Pratama, Maryam Chaib De Mares, Jan Dirk van Elsas

**Affiliations:** Department of Microbial Ecology, Microbial Ecology—Groningen Institute for Evolutionary Life Sciences, University of Groningen, Groningen, Netherlands

**Keywords:** *Paraburkholderia*, soil, prophages, mobile genetic elements, evolution

## Abstract

The genus *Paraburkholderia* encompasses mostly environmental isolates with diverse predicted lifestyles. Genome analyses have shown that bacteriophages form a considerable portion of some *Paraburkholderia* genomes. Here, we analyzed the evolutionary history of prophages across all *Paraburkholderia* spp. Specifically, we investigated to what extent the presence of prophages and their distribution affect the diversity/diversification of *Paraburkholderia* spp., as well as to what extent phages coevolved with their respective hosts. Particular attention was given to the presence of CRISPR-Cas arrays as a reflection of past interactions with phages. We thus analyzed 36 genomes of *Paraburkholderia* spp., including those of 11 new strains, next to those of three *Burkholderia* species. Most genomes were found to contain at least one full prophage sequence. The highest number was found in *Paraburkholderia* sp. strain MF2-27; the nine prophages found amount to up to 4% of its genome. Among all prophages, potential moron genes (e.g., DNA adenine methylase) were found that might be advantageous for host cell fitness. Co-phylogenetic analyses indicated the existence of complex evolutionary scenarios between the different *Paraburkholderia* hosts and their prophages, including short-term co-speciation, duplication, host-switching and phage loss events. Analysis of the CRISPR-Cas systems showed a record of diverse, potentially recent, phage infections. We conclude that, overall, different phages have interacted in diverse ways with their *Paraburkholderia* hosts over evolutionary time.

## Introduction

The interaction between bacterial hosts and bacteriophages (phages) has been intensively studied (reviewed in Salmond and Fineran, 2015). A known consequence of such interaction, which is mainly driven by lysis (fitness) pressure from phages, is bacterial diversification (Canchaya et al., 2004). This diversification is the result of an evolutionary arms race, where bacteria and phages constantly develop new attack-defense strategies to impede partner's mechanisms (Stern and Sorek, 2011; Wang et al., 2016).

Since their discovery, phages have fundamentally changed our traditional view—from a simple parasitic interaction to co-evolution dynamics—of bacterial hosts and phages (Canchaya et al., 2004; Obeng et al., 2016). As the most abundant entities in the biosphere, phages commonly outnumber bacteria by at least one order of magnitude; they are estimated to infect about 10^23^ to 10^25^ bacterial cells every second in ocean ecosystems (Keen et al., 2017). Considerable numbers of phages have been shown to be present in bacterial genomes. In fact, integrated phages (prophages) are at the heart of bacterial diversification processes, e.g., in *Escherichia coli* (Lawrence and Ochman, 1998; Ohnishi et al., 2001; Touchon et al., 2016), *Streptococcus agalactiae. S. pyogenes, Salmonella* sp., *Listeria innocua*, and *L. monocytogenes* (Canchaya et al., 2004). Phages play essential roles in the life of their hosts, from the individual to the population level. For instance, the evolution of bacterial pathogenicity (Brüssow et al., 2004), human health (Manrique et al., 2016), and global nutrient cycling in ocean ecosystems are all affected by phage activities (Roux et al., 2016).

Phylogenetic approaches (in particular co-phylogenetic analyses), have been used to answer questions with respect to the co-evolution of tightly associated members of a community, such as viruses and their hosts (Geoghegan et al., 2017). Given the evolutionary timeline of these relationships, it is expected that congruence, or phylogenetic similarity, is detected from both partners. Congruence is unlikely to occur as a process of simple co-speciation (the process of speciation of one species in response to another one). It is entangled with other evolutionary mechanisms, such as duplications, host-switching, losses and failure to diverge (Conow et al., 2010; Hutchinson et al., 2017). To unravel the co-evolutionary scenario between prophages and their *Paraburkholderia* hosts, two approaches have been applied. First, global-fit/distance-based approaches address the congruence between the phylogenies of the associated organisms and evaluate the dependency of the phage phylogeny upon the host's tree (Hutchinson et al., 2017). The second approach is an event-based approach. This approach considers, for example, duplication, host-switching, and losses, in order to assess the co-evolutionary events (Conow et al., 2010).

Despite offering, in some cases, fitness advantages to their bacterial hosts, phages often provide a “burden” to host functioning that may lead to host cell death by lysis. Clustered regularly interspaced short palindromic repeats (CRISPRs) and their associated proteins (Cas) provide bacteria with protection against invading genetic elements such as phages and plasmids (Makarova et al., 2015). The CRISPR-Cas system is able to acquire short (26–72 bp) sequences of foreign DNA, named proto-spacers, and flank these sequences with proto-spacer-adjacent motifs (PAMs) to make spacers, integrating these into so-called CRISPR arrays (Makarova et al., 2015). The CRISPR-encoded RNA then guides complexes of Cas proteins, which recognize and cleave incoming foreign genetic material at specific sites, preventing further infection. Thus, CRISPR spacers are protective “immune” functions, that can provide insight into the history of bacterial host/phage interplays (Sun et al., 2015). Such interplays spur the diversity of phages (Shmakov et al., 2017), as shown by analyses of the sequences of CRISPR spacers that have little or no homology to any known sequences (Edwards et al., 2016).

Prophages can make up to about 20–30% of the size of bacterial genomes (Casjens, 2003). A previous study has shown the presence of inducible prophage sequences in the genome of the fungal-interactive *Paraburkholderia terrae* strain BS437 (Pratama and van Elsas, 2017). *Paraburkholderia* species inhabit the mycosphere (the soil surrounding fungal hyphae), where frequent exchange of genes across the local microbes is possible (Haq et al., 2014; Zhang et al., 2014). We hypothesized that, by analyzing the presence of phages and CRISPR-Cas systems (especially CRISPR spacers) in the genomes of *Paraburkholderia* spp., we will unearth the evolutionary record of recent phage infections and shed light on the dynamic arms race interaction between the bacterial host and its phages. Here, we address the following key questions: to what extent does the presence of prophages and their distribution affect the diversity and diversification of *Paraburkholderia* spp.? To what extent did co-evolution occur between these partners? And does the presence of CRISPR arrays in bacterial genomes reflect this interaction in natural systems?

## Materials and methods

### Bacterial growth conditions, genome sequencing, and assembly

The 12 newly-sequenced *Paraburkholderia* sp. genomes (*P. terrae* strains BS001, BS007, BS110, BS437, and DSM 17804^T^; *P. phytofirmans* strains BS455, PsJN, BIFAS53, and J1U5; *P. hospita* DSM 17164^T^*, P. caribensis* DSM 13236^T^, and *Paraburkholderia* sp. MF2-27) were used. Strains BS001, BS007, BS110, BS437, BS455, BIFAS53, and J1U5 have been previously isolated in our group (Warmink et al., 2011; Nazir et al., 2012; Pratama et al., 2017). All strains were grown aerobically in Luria-Bertani (LB) broth at 28°C (180 rpm, shaking, overnight). The genomic DNA of the overnight cultures was then extracted using a modified (UltraClean) DNA isolation kit (MOBio Laboratories Inc., Carlsbad, CA, USA). The modification consisted of adding glass beads to the cultures in order to spur mechanical cell lysis. The extraction method is a rapid way to produce highly pure DNA from bacterial cultures. The extracted DNAs were purified with the Wizard DNA cleanup system (Promega, Madison, USA). The quality and quantity of the extracted DNAs were assessed using electrophoresis in 1% agarose gels.

The genomic DNAs of *P. terrae* strains BS001, BS110, BS007, and BS437 and of *P. phytofirmans* strains BS455, PsJN, BIFAS53, and J1U5 were sequenced on the Illumina HiSeq2000 platform by LCG Genomics (Berlin, Germany) (Nazir et al., 2012; Pratama et al., 2017). Those of *P. terrae* strain DSM 17804^T^, *P. hospita* DSM 17164^T^*, P. caribensis* DSM 13236^T^ and *Paraburkholderia* sp. MF2-27 were sequenced by PacBio sequencing (Pacific Biosciences) at the Leibniz Institut DSM (Deutsche Sammlung von Mikro-organismen und Zellkulturen GmbH, Braunschweig, Germany; see Table 1 for species used in this study).

**Table 1:** *Paraburkholderia* species used in this study.

**Species**	**Strains**	**Accession number^a^**	**Assembly level**	**#contigs^b^**	**Genome size (bp)**	**GC%**	**Habitat note^c^**	**#PP^d^**	**PP genome (Kb)^e^**
*P. fungorum*	NBRC 102489	NZ_BAYC0100001-NZ_BAYC0100124	Contig	124	8,696,214	61.84	Fungal-associated (N-P)	1	13.2
*P. sordidicola*	LMG 22029	NZ_FCOC01000001-NZ_FCOC01000072	Contig	72	6,848,654	60.16	Fungal-associated (N-P)	2	47.9
*P. terrae*	BS001	AKAUA01000000	Contig	330	11,294,072	61.82	Fungal-associated (N-P)	1	36.7
*P. terrae*	BS007	NFVE00000000	Contig	788	11,025,273	61.89	Fungal-associated (N-P)	1	20.7
*P. terrae*	BS110	NFVD00000000	Contig	658	11,178,172	61.83	Fungal-associated (N-P)	2	34.5
*P. terrae*	BS437	NFVC00000000	Contig	843	11,303,071	61.78	Fungal-associated (N-P)	2	76.3
*P. terrae*	DSM 17804^T^	CP02611-CP02614	Complete	4	10,062,489	61.92	Fungal-associated (N-P)	1	25
*P. terrae*	NBRC 100964	NZ_BBJK01000001-NZ_BBJK01000247	Contig	247	9,925,782	61.96	Forest soil (N-P)	1	25
*P. hospita*	DSM 17164^T^	CP026105-CP026110	Complete	6	11,527,706	61.79	Soil (N-P)	2	40.7
*P. caribensis*	DSM 13236^T^	CP026101-CP026104	Complete	4	9,032,490	62.58	Soil (N-P)	2	89.9
*P. caribensis*	MWAP64	NZ_CP013102-05	Complete	4	9,032,119	62.58	Vertisol soil (N-P)	2	89.5
*Paraburkholderia* sp.	MF2-27	NP	Complete	7	9,573,839	61.68	Soil (N-P)	9	419.4
*P. ferrariae*	NBRC 106233	NZBAYB01000001-NZBAYB01000097	Contig	97	7,938,642	64.82	Iron ore (N-P)	1	21.4
*P. ginsengisoli*	NBRC 100965	NZ_BBIF01000001-50	Contig	50	6,541,887	63.61	Soil (N-P)	1	33.7
*P. glathei*	KpR1_Mero_10 m	NZ_CCNS1000001-NZ_CCNS1000138	Contig	138	7,492,386	64.73	Acid lateritic (N-P)	4	111.9
*P. oxyphila*	NBRC 105797	NZ_BAYD01000001-NZ_CCNS10000313	Contig	313	10,647,665	64.14	Acidic forest soil (N-P)	2	50.1
*P. phenoliruptrix*	AC1100	NZ_ASXI01000001-NZ_ASXI01000286	Contig	286	7,811,030	63.14	Soil (N-P)	1	33.8
*P. xenovorans*	LB400	NC_007951-NC_007953	Complete	3	9,731,138	62.63	Soil (N-P)	2	85.4
*P. kururiensis*	M130	NZ_ANSK01000001-NZ_ANSK01000009	Contig	9	7,128,857	65.02	Aquifer (N-P)	1	27.4
*P. zhejiangensis*	CEIB S4-3	NZ_JSBM01000001-NZ_JSBM01000154	Contig	154	7,630,666	62.78	Sludge (N-P)	1	14.8
*P. andropogonis*	ICMP2807	NZ_LAQU01000001-NZ_LAQU01000272	Contig	272	6,065,807	58.99	Sorghum bicolor (P)	1	19.5
*P. bannensis*	NBRC 03871	NZ_BAYA01000001-NZ_BAYA01000102	Contig	102	8,648,774	63.96	Grass-associated (P)	3	98
*P. bryophila*	376MFSha3.1	NZ_KB911034-NZ_KB911065	Contig	32	7,381,819	61.86	Moss-associated (P)	0	0
*P. caledonica*	NBRC 102488	NZ_BAYE01000061-NZ_BAYE01000075	Contig	75	7,282,355	61.97	Plant-assosiated (P)	0	0
*P. graminis*	C4D1M	NZ_ABLD01000001-NZ_ABLD01000070	Contig	70	7,477,263	62.87	Plant-assosiated (P)	2	50.3
*P. grimmiae*	R27	NZ_JFHE01000001-NZ_JFHE01000160	Contig	160	6,661,774	63.04	*Xerophilous* moss (P)	1	33.6
*P. heleia*	NBRC 101817	NZ_BBJH01000001-NZ_BBJH01000110	Contig	110	8,007,470	64.61	Aquatic plant (P)	1	23.6
*P. mimosarum*	NBRC 106338	NZ_BBJJ01000001-NZ_BBJJ01000381	Contig	381	8,491,217	63.8	Plant root (P)	4	99.3
*P. nodosa*	DSM 21604	NZ_JAFA01000001-NZ_JAFA01000133	Scaffold	113	9,627,966	64.07	Root nodules (P)	4	119.3
*P. phymatum*	STM815	NC_010622, NC_010623, NC_010625, NC_010627	Complete	4	8,676,562	62.29	Root legume nodule (P)	5	138.3
*P. phytofirmans*	PsJN	NC_010681, NC_010676, NC_010679	Complete	3	8,214,658	62.29	Plant-assosiated (P)	1	63
*P. phytofirmans*	BS455	NP	Contig	209	8,859,905	62.15	Plant-assosiated (P)	2	64.8
*P. phytofirmans*	BIFAS53	NP	Contig	222	8,267,758	61.63	Plant-assosiated (P)	1	13.3
*P. phytofirmans*	J1U5	NP	Contig	689	10,330,795	61.09	Plant-assosiated (P)	2	23.4
*P. sacchari*	LMG 19450	NZ_JTDB01000001-NZ_JTDB01000112	Contig	112	7,263,741	64.04	Sugarcane (P)	2	27.5
*P. sprentiae*	WSM 5005	NZ_KI421529-34 NZ_AXBN01000062, NZ_AXBN01000035	Contig	8	7,761,063	63.18	Root legume nodule (P)	1	11.2
*B. pseudomallei*	K96243	NZ_CP009537-NZ_CP009538	Complete	2	7,247,614	68.06	Human pathogenic (N-P)	4	115.3
*B. cenocepacia*	J2315	NC_011000-NC_011003	Complete	4	8,055,782	66.9	Soil (P)	4	128.5
*B. glumae*	PG1	NZ_CP002580, NZ_CP002581	Complete	2	7,896,538	68.77	Plant pathogenic (P)	2	50

a NP, Not yet published;

b #, number;

c The habitat of the Paraburkholderia were manually checked and thus crossed-checked from the GOLD database version October 2017 (see section Materials and Methods);

d PP, prophages;

e total amount of prophage sequence in genome;

T *type strain*.

### Bacterial genome data retrieving

**Figure 1:**
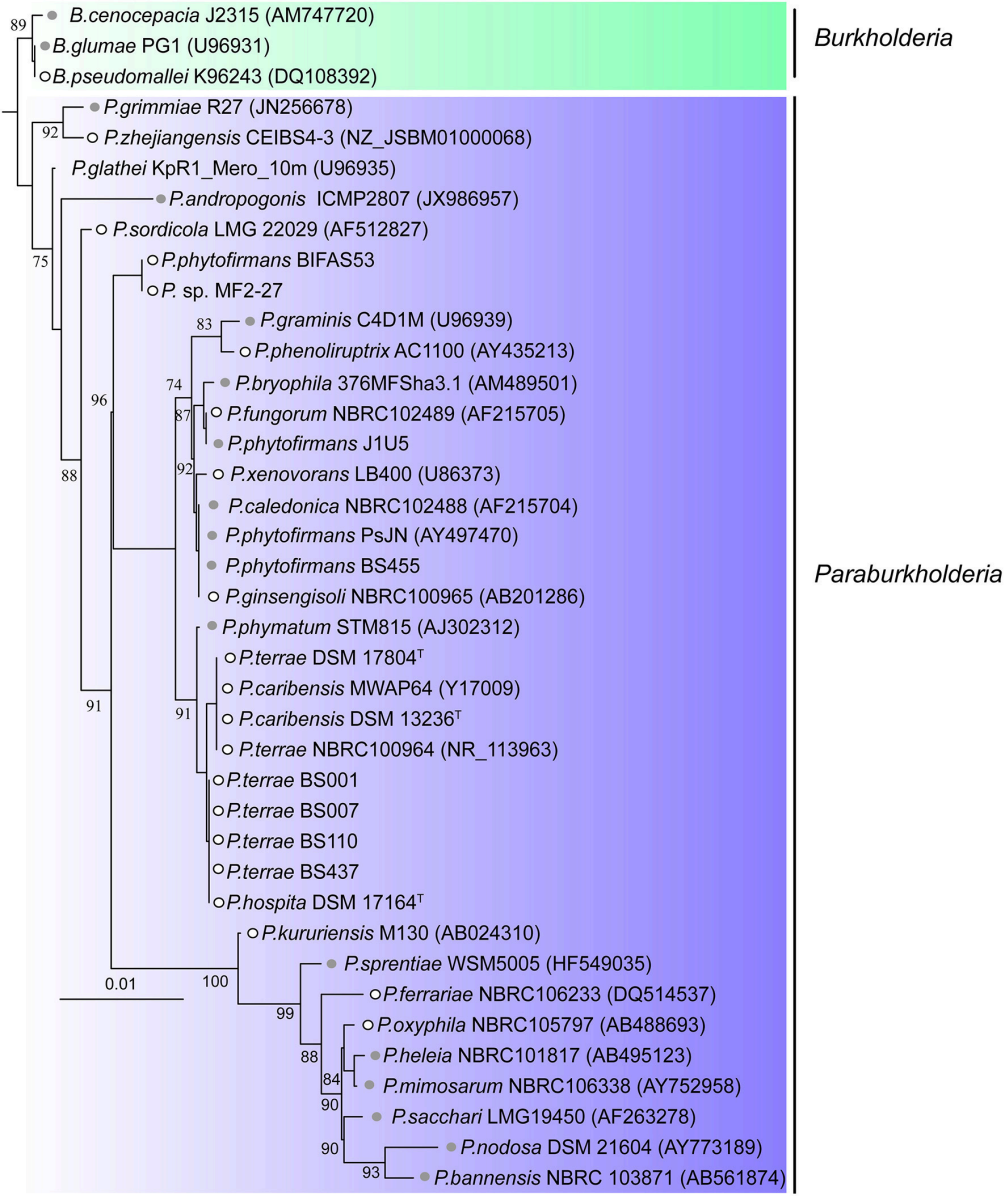
Phylogeny of the 36 *Paraburkholderia* and three *Burkholderia* species used in this study. The 16S rRNA genes of the 39 bacteria were aligned using the SINA (Pruesse et al., 2007) and MAFFT (Katoh et al., 2002) alignment tools. The alignment was edited using Gblocks (Talavera et al., 2007). A maximum likelihood based phylogenetic tree was then constructed using FastTree (Price et al., 2009) and the tree (midpoint-rooting) was visualized using iTOL (Letunic and Bork, 2016). Gray circles represent “plant-associated” *Paraburkholderia* species, while white circles represent “non-plant-associated” *Paraburkholderia* species. See section Materials and Methods for explanation of plant- vs. non-plant-association.

Initially, we entered the *Burkholderia* database (http://www.burkholderia.com/strain/download, last accessed in March 2017), yielding a total of 1,185 strains (containing 123 complete genomes and 1,062 drafts of *Burkholderia* genomes). We then selected the recently-named genus *Paraburkholderia*, primarily containing 62 environmental species that are non-pathogenic (Sawana et al., 2014). Among the selected genomes, we found 24 species with complete genomes in the database. We included three outgroup species (i.e., *Burkholderia glumae, B. cenocepacia*, and *B. pseudomallei*) in the initial prophage identification analysis. A total of 36 genomes of *Paraburkholderia* spp. and three genomes of *Burkholderia* were thus used in this study (see Table 1 and Figure 1). The predicted habitats (“P”—plant-associated and “N-P”—Non-plant-associated) of the *Paraburkholderia* species were taken from literature data and then crossed-checked using the GOLD database, version October 2017. Here, what we call plant-associated vs. non-plant-associated (including fungal-interactive) *Paraburkholderia* species might not strongly reflect the true nature of these species, as some fungi can occur in the rhizosphere and so also be plant-associated.

### Phylogenetic analysis and genome comparisons

Prophage phylogenetic trees were generated using selected concatenated phage signature genes (i.e., capsid, portal, tail tape, and terminase), next to the individual phylogenies of those genes. The predicted proteins were aligned with MUSCLE (Edgar, 2004). The 16S rRNA genes of the *Paraburkholderia* strains were used to align with SINA (Pruesse et al., 2007) and MAFFT alignment tools (Katoh et al., 2002). Both phage and host gene alignments were edited using Gblocks (Talavera et al., 2007), with default parameters. Then, maximum likelihood phylogenetic trees were constructed using FastTree (Price et al., 2009) and these (midpoint-rooting) were visualized using iTOL (Letunic and Bork, 2016). Furthermore, genome comparison percentages were generated using BLAST + 2.4.0 (tBLASTx with cut-off value 10^−3^) and map comparison figures created with Easyfig (Sullivan et al., 2011).

### Detection of prophage and CRISPR-Cas arrays (spacers)

Prophage regions were detected in the bacterial genomes using PHAST (Zhou et al., 2011). PHAST uses current publicly available viral databases, such as “NCBI phages and viruses,” to identify prophage position, length, boundaries, number of genes and attachment sites, such as tRNA sites. The completeness of the identified prophage regions was determined based on scores that consider three scenarios: (i) complete prophage regions contain only genes for known phage proteins and the joint proteins conceptually allow building a functional phage, (ii) incomplete prophage regions are defined as having >50% genes for proteins that are predicted to be of prophage nature, and (iii) questionable prophage regions are defined as having <50% genes for proteins predicted to be of prophage nature. Three other criteria were used to further define prophage regions, those are (i) regions with sizes below 10 Kb, (ii) regions lacking genes for phage core/hallmark proteins (e.g., tail protein, capsid/head protein, terminase and integrase) and (iii) regions with > 25% insertion sequence (IS) elements (that is, short DNA sequences that act as simple transposable elements) were discarded (Bobay et al., 2013). The resulting manually curated prophages, with genes for structural proteins, replication, recombination, and lysis proteins, were further analyzed.

The analyses of CRISPR-Cas arrays (repeat and spacers) were performed on the genomes of all known *Paraburkholderia* spp. from CRISPRdb (Grissa et al., 2008), which were downloaded to our local server. CRISPRFinder (http://crispr.i2bc.paris-saclay.fr/Server/) was then used to identify any spacers from bacterial genomes that are not in the database. Thus, Cas proteins were annotated using CRISPRone (Zhang and Ye, 2017). Manual readings were applied to the identified CRISPR-Cas systems using as criteria: (i) Regions with more than three repeats and two spacers were considered to constitute *bona fide* CRISPR arrays, (ii) CRISPR arrays lacking Cas proteins in the vicinity were kept, as predicted orphan CRISPR arrays (Makarova et al., 2015; Almendros et al., 2016), and (iii) CRISPR-Cas loci lacking CRISPR arrays were discarded. The classification and clustering of CRISPR repeats were analyzed using CRISPRmap, a comprehensive cluster analysis method (based on HMM clustering), which clusters conserved sequence families and potential structural motifs (Lange et al., 2013). To determine the record of past infections by phages and/or other mobile genetic elements (MGEs), all unique spacers were compared against the NCBI (viruses, phages, and plasmid) databases (last accessed: December 2017) using BLASTN (Altschul et al., 1997); the 11-nt word size was used in BLASTN. The results showing highest coverage (≥70%) and identity were considered to represent valid hits. The analyses using BLAST all-vs.-all were carried out by doing BLAST the identified prophage dataset against the spacer dataset. The analyses of proto-spacers were also done using IMG/VR (https://img.jgi.doe.gov/cgi-bin/vr/main.cgi) against their viral spacer and metagenome spacer database (Paez-Espino et al., 2017).

### Prophage-host co-phylogeny analyses

Co-phylogeny analyses were performed using two methods: first, we used PACo (Procrustes approach to co-phylogenetics), which assesses the congruence or evolutionary dependency, of two groups of interacting species using both ecological interaction networks and their phylogenetic history. The analysis (evaluating the distribution of a set of shapes) produced superimposition plots (illustrating the correspondence coordinates of divergences between lineages, or patristic distances), in which the global-fit of prophage phylogenies onto the hosts is shown. The contribution of each individual host-prophage association to the global-fit is also evaluated (Hutchinson et al., 2017). The second approach is an event-based approach, which evaluates the combination of events of co-speciation, duplication, duplication with host switching, loss and failure to diverge, using Jane 4 (Conow et al., 2010). The default settings used were co-speciation = 0, duplication = 1, duplication and host switching = 2, sorting = 1, failure to diverge = 1. To find the best solution, while minimizing the cost of co-evolutionary events, a genetic algorithm (GA) that relies on bio-inspired operators, in this case randomized host, and prophage trees, was applied. The GA algorithm consists of population (the number of different solutions being considered at each iteration of the algorithm) and generation (the number of iterations performed by the algorithm as it seeks a good reconstruction of the parasite tree onto the host tree). These were set at 1,000 and 10, respectively (Conow et al., 2010). The statistical significance of the reconstructions was evaluated with 100,000 random tip mapping permutations.

### Statistical analysis

Statistical analysis of the prophage distribution was performed using RStudio (Integrated Development for R. RStudio, Inc., Boston, MA URL http://www.rstudio.com/). A Shapiro-Wilk test was used for testing the normality of the data distribution (Razali and Wah, 2011) and a Kolmogorov-Smirnov test to assess the significance of differences for non-normally-distributed data. For all statistical significance tests, the significance level was set to 95%.

## Results

### Identification and distribution of prophages across selected *Paraburkholderia* genomes

**Figure 2:**
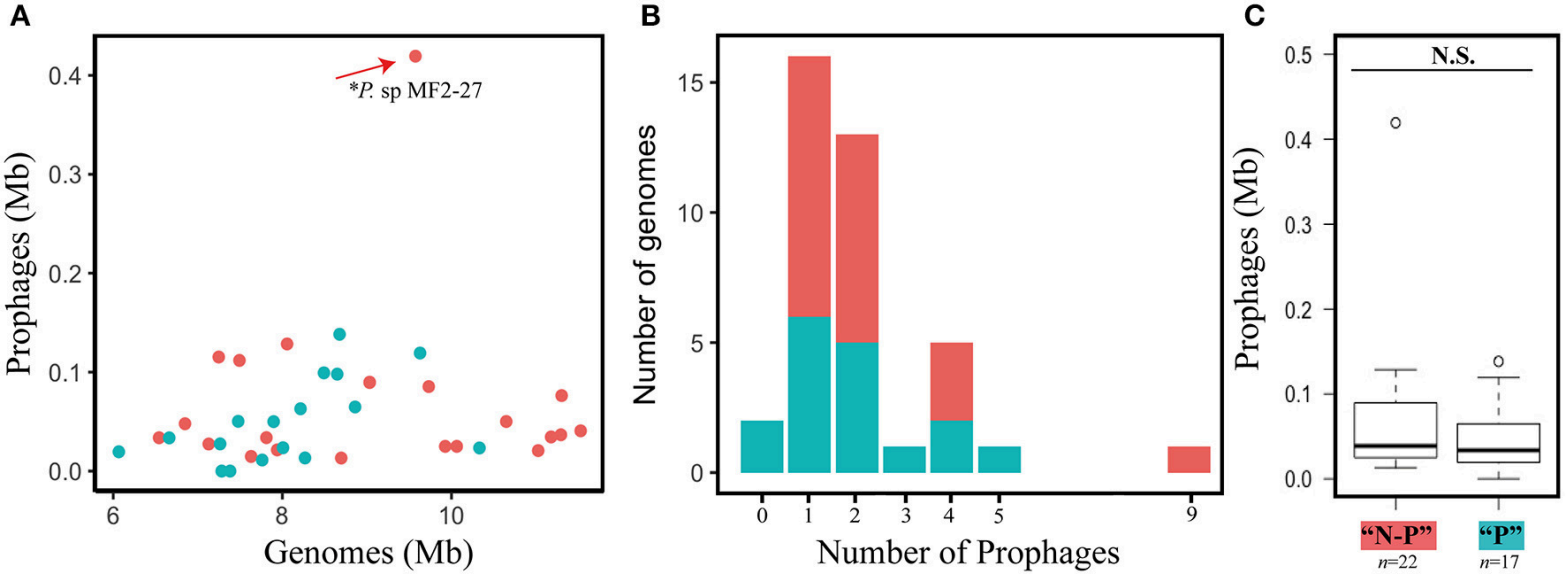
Prophage distribution. **(A)** Prophage size depicted as related to host genome size. Dots in blue correspond to plant-associated *Paraburkholderia* spp., while red dots correspond to non-plant-associated *Paraburkholderia* spp. ^*^Represents the outlier *Paraburkholderia* sp. MF2-27. **(B)** Distribution of the number of prophages per genome. **(C)** Box-plot of the distribution of size of prophage genomes (Mb) of plant-associated and non-plant-associated *Paraburkholderia* spp. (Kolmogorov-Smirnov test, N.S.–not significant, ρ = 0.3454). See section Materials and Methods for explanation of “P”–plant- vs. “N-P” non-plant-association.

Prophages and prophage-derived elements were identified using the criteria stated in section Materials and Methods. A total of 105 genomic regions meeting these criteria were found before manual curation. Following removal of sequences <10 Kbp, those lacking genes for phage tail, capsid/head, terminase and integrase proteins and those with > 25% of ISs, we ended up with a total of 79 prophages in the genomes that were analyzed (Table 1). These included the genomes of the three *Burkholderia* species used as an outgroup (Figure 1). Most of the prophages (75%) identified consisted of “remnants” of past infection events, as evidenced by the loss of essential phage genes (e.g., structural and replication genes). This finding is consistent with the deletion bias theory (Bobay et al., 2014) and with the idea that these phage insertions represent ancient events (Hendrix et al., 2001). We detected prophages in most of the selected *Paraburkholderia* strains and in the three outgroup *Burkholderia* (94.87%, *n* = 37), the exceptions being *P. bryophila* 376MFSha3.1 and *P. caledonica* NBRC 102488. The number of identified prophages per genome ranged from one to nine, with most of the *Paraburkholderia* species (27%, *n* = 10) harboring just one prophage region meeting the criteria. Remarkably, one outlier genome, that of *Paraburkholderia* sp. MF2-27, contained nine prophage regions, which stood in sharp contrast to the number present in all other genomes (range 16.8 to 62 Kb; Figure 2). The genome sizes of the identified prophages ranged from 11.5 to 419 Kb, contributing up to 4% of the genomes of the *Paraburkholderia* strains. The G+C contents of the prophages were on the average 60.5% (ranging from 56-66%), which was invariably below that of their hosts (average = 63%; ranging from 58.88 to 67.88%). No significant differences observed (Kolmogorov-Smirnov, N.S.—not significant ρ = 0.3454) between the prophage genome sizes in the genomes of the “plant-associated” vs. the “non-plant-associated” *Paraburkholderia* species (Figure 2C).

Here, we further focus on the 25% more recently acquired prophages, defined on the basis of their possession of full gene sets for phage particle assembly (e.g., genes for tail, capsid genes), replication, recombination and lysis proteins. Thus, we selected a total of 26 prophages, identified in the genomes of 17 *Paraburkholderia* strains, for further analysis (Table 2).

**Table 2:** Complete prophages detected in this study^a^.

***Paraburkholderia* species**	**Strain**	**Phage^b^**	**Position**	**Phage genome (Kb)**	**#ORFs**	**GC%**	**GC% host**
*Paraburkholderia terrae*	BS437	ϕ 437	2,544,472–2,598,070	53.50	90	60.31	61.78
*P. terrae*	DSM 17804^T^	ϕ Pt17804	2,002,606–2,027,700	25.00	33	63.40	61.92
*P. terrae*	NBRC 100964	ϕ PtNBRC	5,729,452–5,754,546	25.00	33	63.40	61.96
*P. bannensis*	NBRC 103871	ϕ Pban1	3,276,307–3,300,593	24.20	30	62.42	63.96
″	NBRC 103871	ϕ Pban2	5,677,339–5,724,895	47.50	74	61.72	*″*
*″*	NBRC 103871	ϕ Pban3	7,436,273–7,462,669	26.30	34	62.60	*″*
*P. graminis*	C4D1M	ϕ Pgram1	955,225–978,978	23.70	29	65.20	62.87
*P. heleia*	NBRC 101817	ϕ Phele1	3,485,522–3,509,193	23.60	33	62.20	63.80
*P. nodosa*	DSM 21604	ϕ Pnodo2	1,296,160–1,332,178	36.00	53	63.40	64.07
*P. phymatum*	STM816	ϕ Pphym1	307,492–351,536	44.00	61	61.30	62.29
*″*	STM816	ϕ Pphym2	1,791,289–1,817,645	26.30	31	61.72	*″*
*P. phytofirmans*	PsJN	ϕ PphytPsJN	1,279,270–1,342,364	63.00	72	59.76	62.29
*P. phytofirmans*	BS455	ϕ Pphyt455	2,101,019–2,146,837	45.80	52	60.10	62.15
*P. caribensis*	DSM 13236^T^	ϕ Pcari1DS	1,961,208–2,006,816	45.60	54	59.40	62.58
*″*	DSM 13236^T^	ϕ Pcari2DS	2,525,737–2,570,108	44.30	53	58.92	*″*
*P. caribensis*	MWAP64	ϕ Pcari1MW	1,580,430–1,624,801	44.30	53	58.91	62.58
*″*	MWAP64	ϕ Pcari2MW	2,142,810–2,188,076	45.20	53	59.40	*″*
*P. glathei*	KpR1_Mero_10 m	ϕ Pgla2	57,502–85,239	27.70	32	63.40	64.73
*P. oxyphila*	NBRC 105798	ϕ Poxy3	8,352,385–8,382,948	30.50	37	61.10	64.14
*Paraburkholderia* sp.	MF2-27	ϕ Psp20	1,138,433–1,191,247	52.80	63	63.62	61.68
*″*	MF2-27	ϕ Psp21	511,009–568,811	57.80	67	64.86	*″*
*″*	MF2-27	ϕ Psp31	2,251,314–2,295,384	44.00	52	64.20	*″*
*″*	MF2-27	ϕ Psp41	3,018,362–3,055,180	36.80	42	60.60	*″*
*″*	MF2-27	ϕ Psp51	4,016,573–4,078,604	62.00	79	64.12	*″*
*″*	MF2-27	ϕ Psp61	4,574,984–4,630,929	55.90	69	63.30	*″*
*P. xenovorans*	LB400	ϕ Pxeno2	1,498,760–1,552,105	53.30	93	61.70	62.63

a *PHAST analysis was used to identify prophage regions; this analysis was based on current viral databases. PHAST identifies prophage regions based on criteria such as length, boundaries, number of genes and attachment sites (see section Materials and Methods for details)*.

b *The naming of Paraburkholderia sp. MF2-27 phages was as follows: the first number represents the number of phage regions and the second number the contig number; “, same genome*.

Prophages often offer lysogenic conversions that advance their hosts' fitness. Here, we found some potential moron genes, on the basis of these being not essential for phage reproduction, in these complete phages (Supplementary Table 1). We found high identity (70–83% identity and 100% coverage) of a DNA adenine methylase gene in several prophages, i.e., ϕPphytPsJN, ϕPphyt455, ϕPcari1DS, ϕPcari2DS, ϕPcari1MW, ϕPcari2MW and ϕPglat2. However, most (i.e., 13.33–71.15%) of the genes in these prophages remained hypothetical (Supplementary Table 1), so it is possible that these phages constitute a repertoire of novel genes that may enhance host fitness.

### Prophage phylogenies and genomic analyses

**Figure 3:**
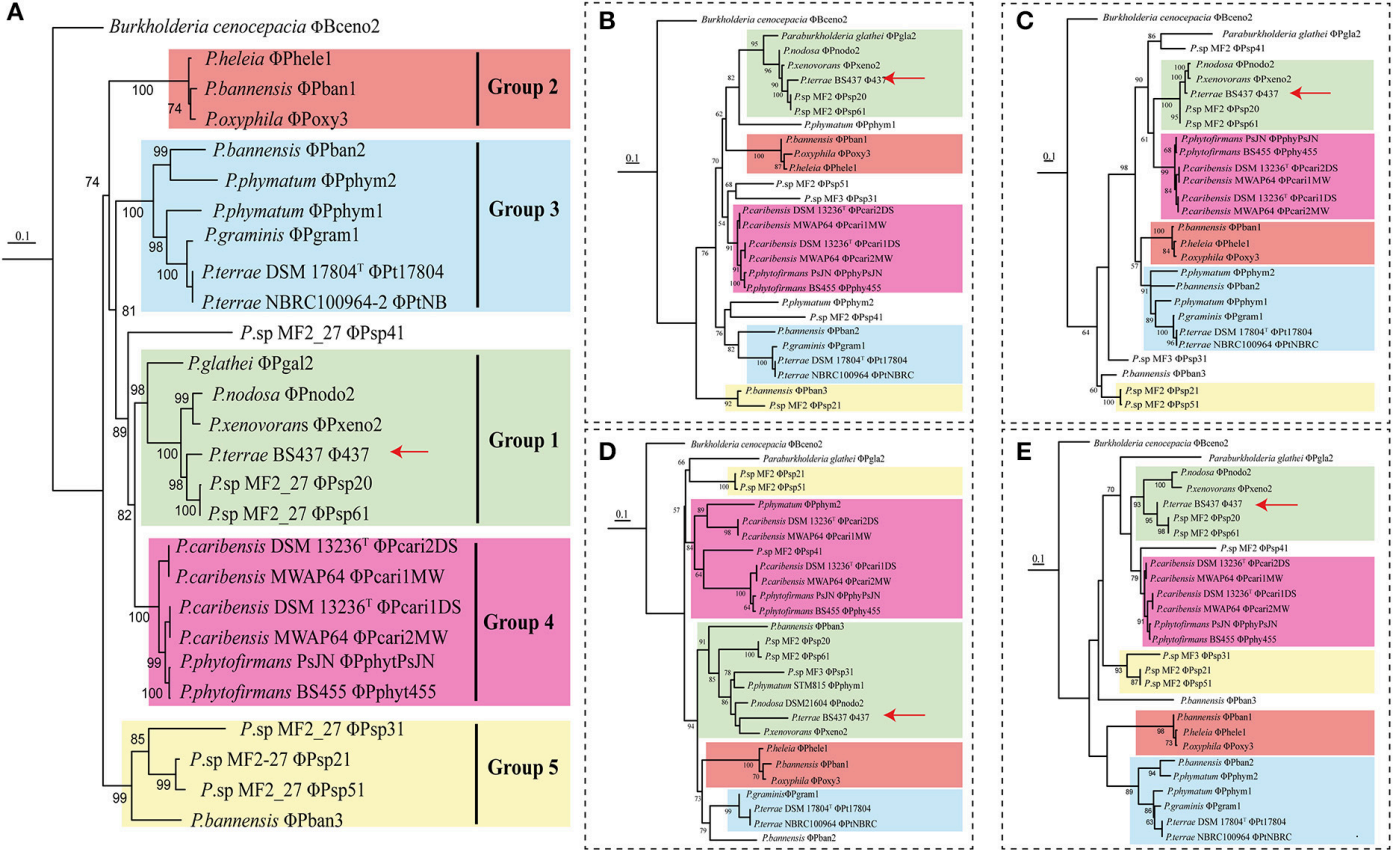
Complete prophage relatedness trees based on **(A)** concatenated phage signature (capsid, portal, tape, and terminase) genes and individual phage genes, i.e., **(B)** capsid, **(C)** portal, **(D)** tape, and **(E)** terminase. The translations of concatenated and individual signature genes were aligned with MUSCLE (Edgar, 2004) and edited using Gblocks (Talavera et al., 2007). Maximum likelihood phylogenetic trees were constructed using FastTree (Price et al., 2009) and trees (midpoint-rooting) were visualized using iTOL (Letunic and Bork, 2016). Colors represent consistent groupings of the prophages. Red arrows indicate phage ϕ437.

**Figure 4:**
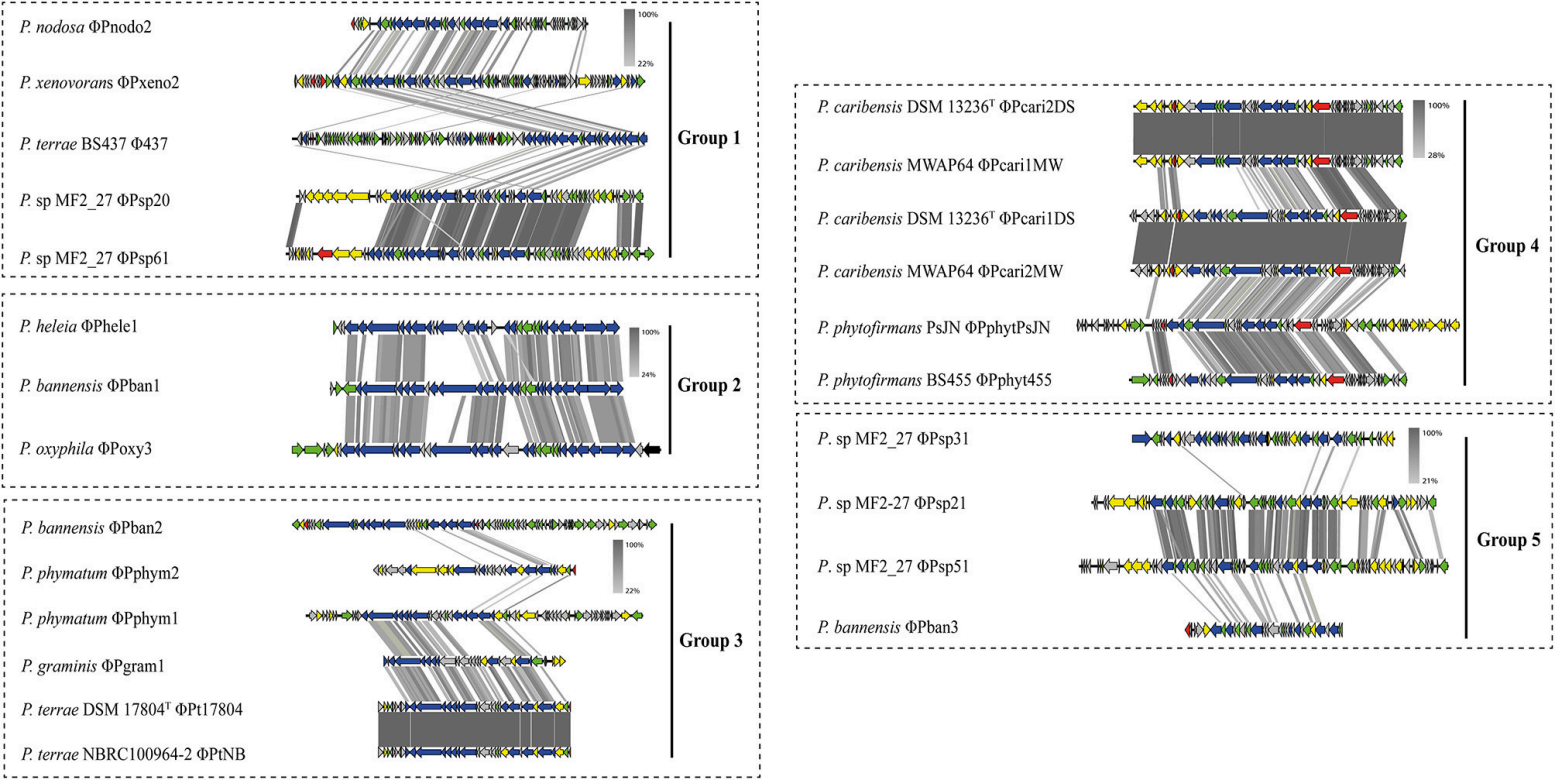
Synteny analysis of complete prophage genomes. The groupings of the prophages are based on the phylogenetic tree constructed with the concatenated phage signature (i.e., capsid, portal, tape, and terminase) genes. Red arrows: phage lysis and lysogeny genes; blue arrows: phage structural genes (tail, capsid, and fiber); green arrows: replication, recombination, repressor, and phage related genes; gray arrows: hypothetical proteins; yellow arrows: non-phage or possible moron genes. Comparison percentage was generated using BLAST + 2.4.0 (tBLASTx with cutoff value 10^−3^) and map comparison figures were created with Easyfig (Sullivan et al., 2011) as indicated in section Materials and Methods. Gene similarity percentage is indicated in gray-scale bars.

To better understand the evolutionary trajectory of the selected prophage regions (Table 2), we built phylogenetic trees based on concatenated as well as single phage signature genes. For these, we selected the genes for phage capsid, portal, tape and terminase proteins (Figure 3). Despite the high divergence across the constructed phage phylogenies, the prophages clustered into five groups, as supported by all phylogenetic trees. Interestingly, the previously-identified *P. terrae* BS437 phage ϕ437 (Pratama and van Elsas, 2017) revealed to be closely related to two prophages from *Paraburkholderia* sp. MF2-27, denoted ϕPsp20 and ϕPsp61, one prophage from *P. xenovorans* (ϕPxeno2) and another one from *P. nodosa* (ϕPnodo2). However, it was distantly related to the other prophages identified in *P. terrae* DSM 17804^T^ and *P. terrae* NBRC 100964. Moreover, the prophages identified in *P. caribensis* strains DSM 13236^T^ and MWAP64, *P. terrae* strains DSM 17804^T^ and NBRC 100964, and *P*. *phytofirmans* strains PsJN and BS455, always clustered in the same group (Figure 3). The grouping among these prophages was consistent with the comparative analyses of the whole genomes of these prophages (see Figure 4 and Supplementary Figure 1), which showed these to be highly syntenous to each other (100, 100, and 38–100% similarity, respectively). The conserved regions in these prophages (i.e., ϕPcari2DS and ϕPcari1MW; ϕPcari1DS and ϕPsp2MW; ϕPt17804 and ϕPtNBRC1; ϕPphytPsJN and ϕPhyt455) included phage genes for structural, replication, recombination and lysis proteins. The genome structures of the 26 complete prophages in *Paraburkholderia* showed different divergences (see Figure 4 and Supplementary Figure 1), which, as we postulate here, were either rearranged via homologous recombinations at short conserved boundary sequences in the phage genomes (Pedulla et al., 2003) or came about by multiple infection events from different phages (Ohnishi et al., 2001). This latter scenario was supported by the fact that the prophages from *Paraburkholderia* sp. MF2-27 (ϕPsp20, ϕPsp21, ϕPsp31, ϕPsp41, ϕPsp51 and ϕPsp61) were highly divergent. However, high levels of synteny were observed in the structures of the prophage genomes, for instance across the replication regions in prophages ϕPsp20 and ϕPsp61, as well as ϕPsp21 and ϕPsp51 (Supplementary Figure 1).

### Host-prophage co-phylogeny analyses

To understand the evolutionary relationships of the 26 identified full prophages and their hosts, we applied the two different methods of co-phylogenetic analysis: PACo (distance-based) and Jane 4 (event-based). The distance-based method showed global-fit values ($m_{XY}^{2}$) between *Paraburkholderia* and their prophages of 0.28 (concatenated genes), 0.31 (capsid), 0.28 (portal), 0.27 (tape), and 0.27 (terminase). To test the robustness of the analyses, we applied 100,000 permutations (*P* < 0.0001) with α = 0.05 as the significance level. The $m_{XY}^{2}$ values were inversely proportional to the topological congruence between the two phylogenies (Balbuena et al., 2013). Therefore, the analyses suggested that the *Paraburkholderia* (host) phylogenetic trees did not predict the topology of the prophage trees, using any of the genes (concatenated, capsid, portal, tape, and terminase; Table 3).

**Table 3:** The number of evolutionary event detected by co-phylogeny analyses with the programs Jane and statistical analysis of global-fit PACo^a^.

**Parameter**	**Jane^a^^,^^b^**							**PACo^c^**
	**S**	**C**	**CS**	**D**	**DS**	**L**	**F**	**${\text{M}}_{\text{XY}}^{2}$**
Concatenated	7,117	32	11	3	12	5	0	0.28
Capsid	2,974	33	11	3	12	6	0	0.31
Portal	5,434	34	10	3	13	5	0	0.28
Tape	4,077	29	13	3	10	6	0	0.27
Terminase	1,845	33	8	3	15	0	0	0.27

a *Jane analyses the combination of co-speciation, duplication, duplication with host switching, loss and failure-to-diverge events in organism-organism co-evolution events, while PACo evaluates the congruence of prophage phylogeny with the host tree and the contribution of each host-prophage link to the congruence (see section Materials and Methods for details)*.

b *S, solution; C, cost; CS, co-speciation; D, duplication; DS, duplication and host switch; L, loss; F, failure-to-diverge; $M_{XY}^{2},$ global-fit value, a measure of the fit of the parasite phylogeny with the host phylogeny*.

c *Statistical analyses of global fit were done using 100,000 permutations (P < 0.0001) at α = 0.05*.

**Figure 5:**
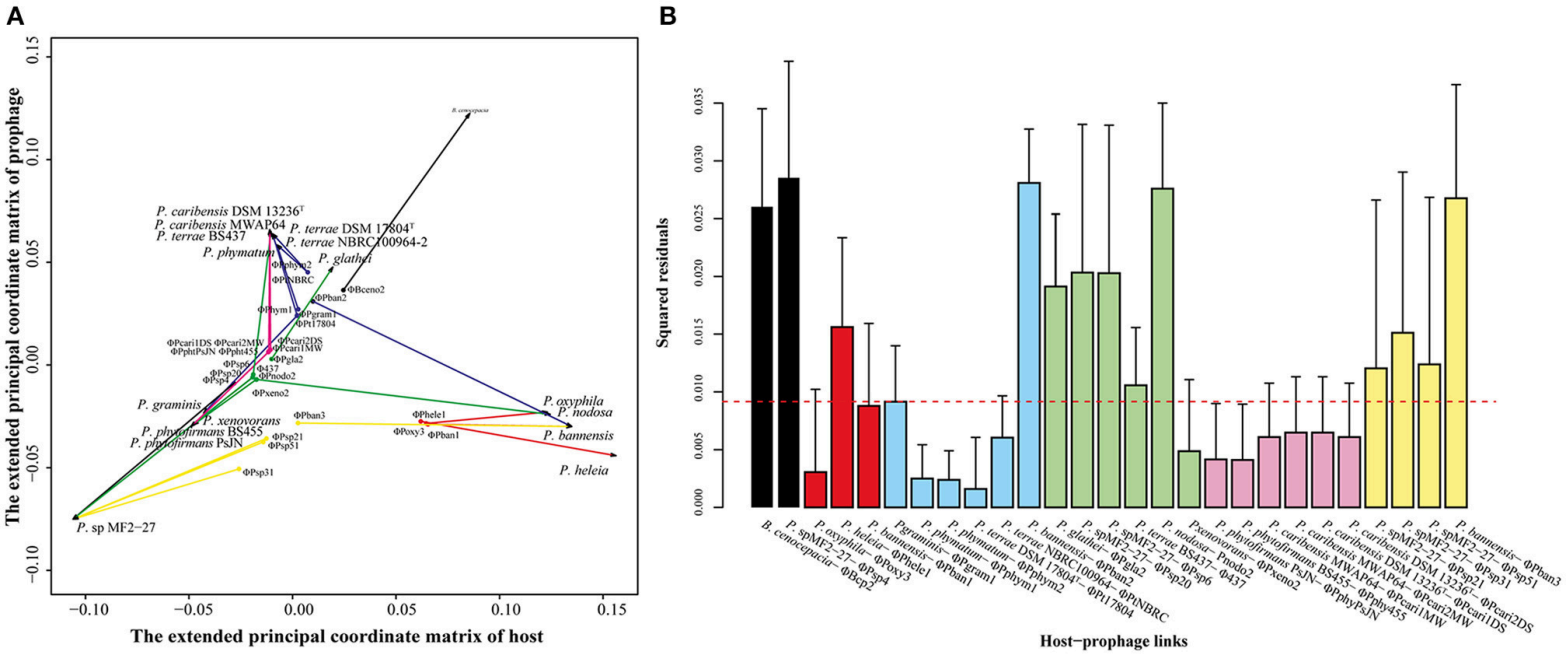
PACo (Procrustes approach to co-phylogeny) results based on *Paraburkholderia* species phylogeny and concatenated prophage phylogeny. **(A)** Procrustean superimposition plot analysis, which minimizes differences between the two partners' principal correspondence coordinates of patristic distances. For each vector, the starting point (black dot) represents the configuration of prophages and the arrowhead the configuration of hosts. The vector length represents the global fit (residual sum of squares), which is inversely proportional to the topological congruence. **(B)** Contribution of each *Paraburkholderia* lineage and their prophages to the general pattern of coevolution. Each bar represents a Jack-knifed squared residual. Error bars represent the upper 95% confidence intervals from applying PACo to patristic distances. Further, the median squared residual value is shown (dashed red line). The colors represent the clusters shown in the prophage phylogeny (see Figure 3).

To identify the extent of the contribution of each prophage host to the co-phylogenetic structure, we then evaluated the procrustean superimposition plots and the Jack-knifed square residual values (Balbuena et al., 2013). The former analysis showed clusters of possible congruencies of the phylogenies of the prophages (concatenated gene tree) and their hosts (Figure 5A and Supplementary Figure 2). Some of these clusters were remarkably close to each other, reflecting the high relatedness with the species phylogeny of their hosts (Figure 1). Moreover, a significant portion of the prophage tree topology presented low squared residual values, specifically for prophages ϕPoxy3, ϕPphym1, ϕPphym2, ϕPt17804, ϕPtNBRC, ϕPxeno2, ϕPphyPsJN, ϕPphy455, ϕPcari1MW, ϕPcari2MW, ϕPcari1DS, and ϕPcari2DS. These lineages thus reflected the tree topologies of their hosts (based on 16S rRNA), suggesting possible co-evolutionary associations (Figure 5B and Supplementary Figure 3).

**Figure 6:**
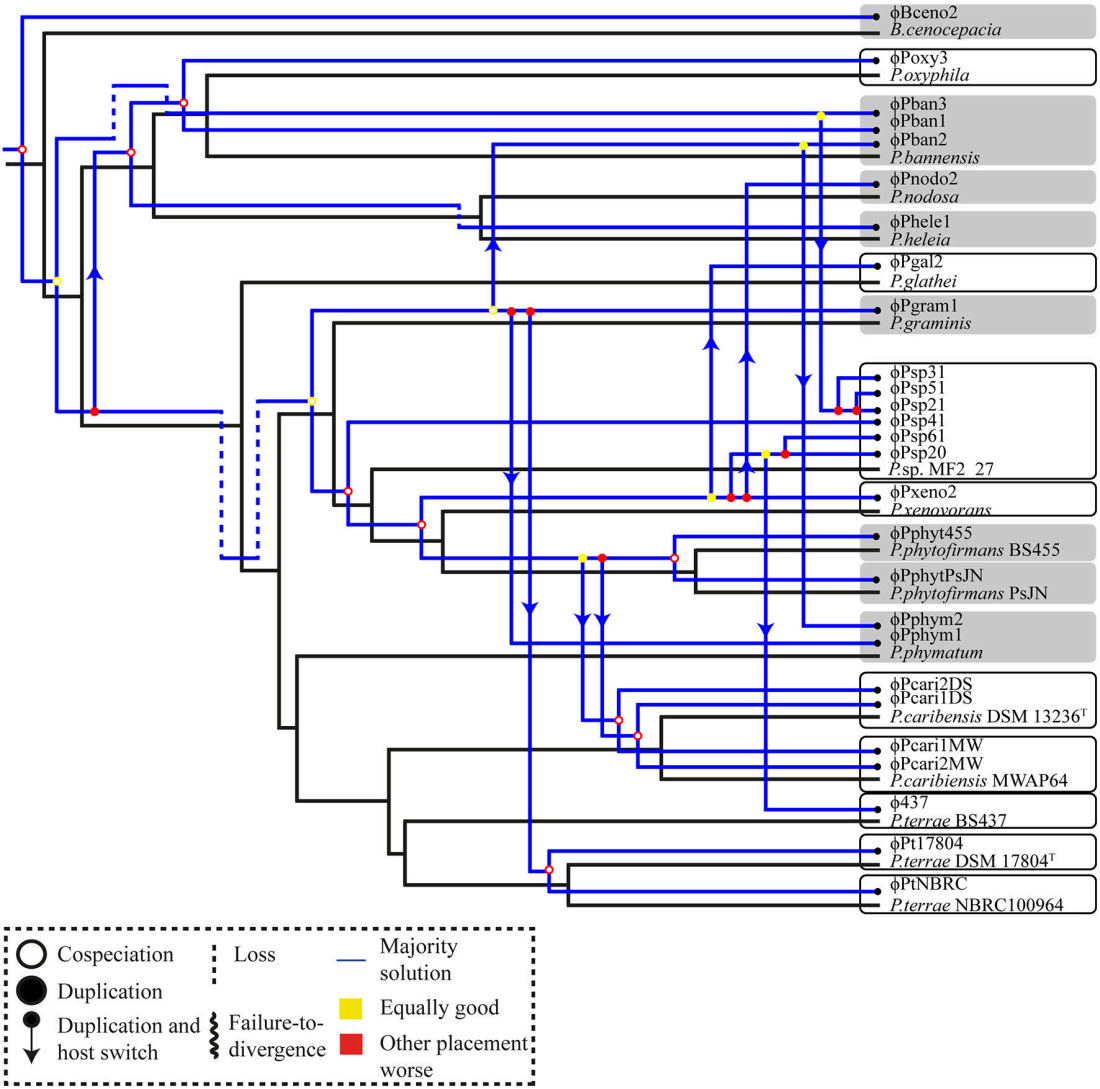
Tanglegram depicting the *Paraburkholderia* (host) species phylogenetic tree in black and the prophage tree in blue. Auxiliary lines connecting the two trees are shown. The event-based method was performed with the default settings for cost regimes (“co-speciation” event = 0; “duplication” event = 1; “loss” event = 1; “duplication then host switching” event = 2) using Jane 4.0 (Conow et al., 2010). All analyses were performed with populations of 1,000 and 10 generations. Gray boxes represent plant-associated *Paraburkholderia* species and white boxes non-plant-associated *Paraburkholderia* species. Jane results showed that host-switching events occurred frequently, next to co-speciation.

The second analysis, which addressed the reconciliation of the prophage-concatenated phylogeny with the host tree, resulted in 31 putative evolutionary scenarios. These included 11 events of co-speciation, three duplications, 12 host-switching and five loss events (Table 3). However, no “failure to diverge” event showed up. Furthermore, three *Paraburkholderia* sp. MF2-27 prophages, i.e., ϕPsp31, ϕPsp51, and ϕPsp21, were predicted to derive from recent host switching by an ancestor of the *P. bannensis* prophage ϕPban3. Additionally, *P. terrae* BS437 phage ϕ437 was found to be derived from recent host switching from *Paraburkholderia* sp. MF2-27 phages ϕPsp20 and ϕPsp61 and *P. nodosa* phage ϕPnodo2 with the common ancestor of prophage ϕPxeno2 (Figure 6 and Supplementary Figure 4). Similar numbers of each of the evolutionary scenarios were obtained from analyses of the individual phage signature genes (see Table 3 and Supplementary Figures 2, 3). Overall, the results show that the evolutionary trajectory of the *Paraburkholderia* prophages is predominantly characterized by host switching, followed by co-speciation events.

### Insight into CRISPR-Cas systems in *Paraburkholderia* genomes

**Figure 7:**
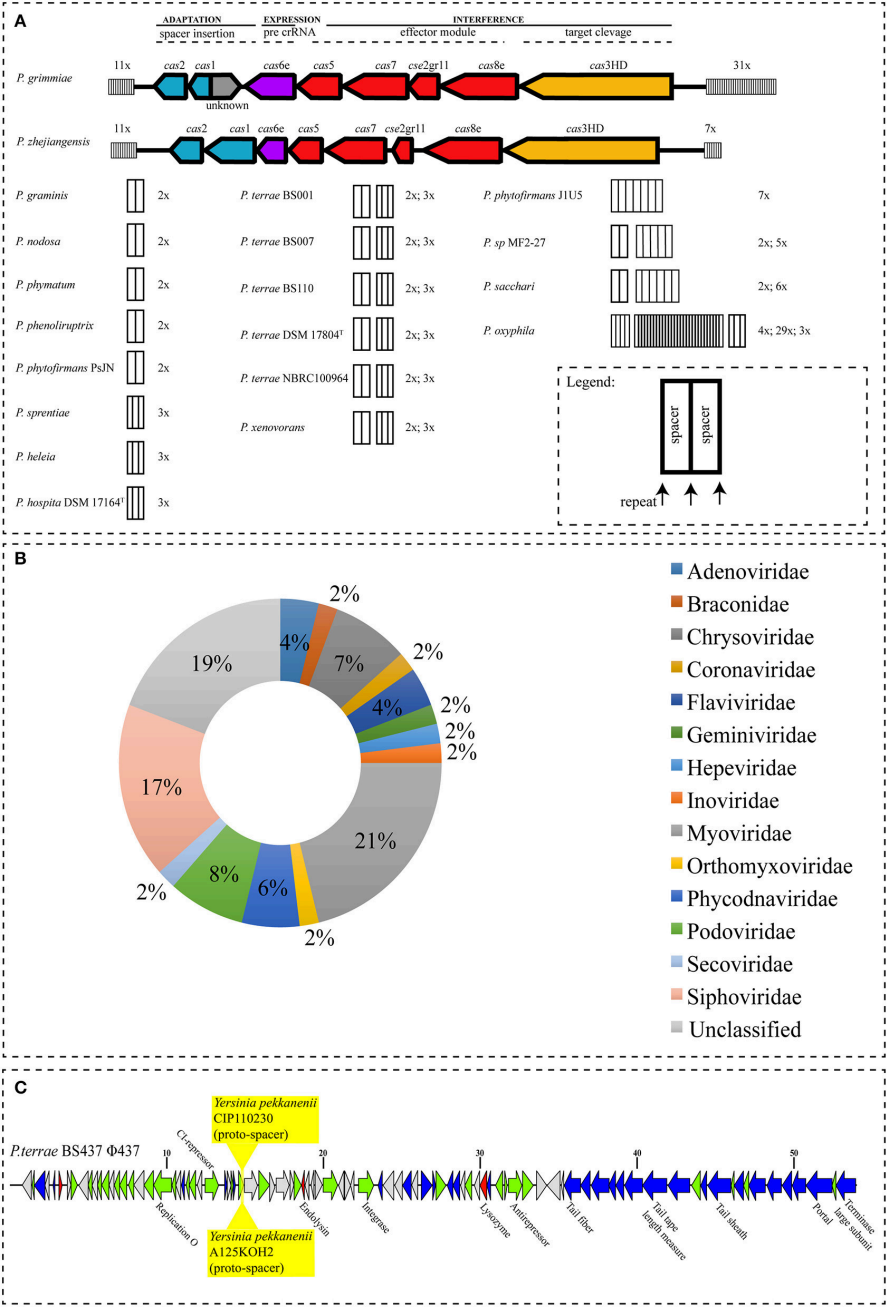
**(A)** CRISPR-Cas systems identified in *Paraburkholderia* species. The *Cas* genes are colored according to their functional category, as in Makarova et al. (2015). CRISPR arrays are represented as black boxes, with black lines representing repeats and white filling denoting spacers. Numbers on top of the CRISPR arrays represent the number of CRISPR-spacers. **(B)** BLAST-based hits of spacer sequences and **(C)** *Yersinia pekkanenii* CIP110230 and *Y. pekkanenii* A125KOH2 proto-spacer hits, analyzed using IMG/VR (Paez-Espino et al., 2017).

To provide insight into the evolutionary history of past infections from bacteriophages in the *Paraburkholderia* strains, we investigated the occurrence of CRISPR-Cas systems, especially CRISPR arrays (spacers). *In silico* analyses were carried out to identify CRISPR-Cas loci and CRISPR arrays, applying strict criteria for detection (see section Materials and Methods). The analyses showed that 55.55% (*n* = 20) of the genomes of the *Paraburkholderia* species harbor identifiable CRISPR-Cas systems (Figure 7A). Two complete systems were found in *P. grimmiae* and *P. zhejiangensis*. These consisted of genes for Cas proteins (*cas*1, *cas*2, *cas*3HR, *cas*5, *cas*6e, *cas*7, *cas8*e, and *cse*2gr11) flanked by two CRISPR arrays with totals of 42 and 18 spacers, respectively (Figure 7A). The complete CRISPR-Cas systems found in these two genomes (*P. grimmiae* and *P. zhejiangensis*) showed the presence of the Cas signature protein *cas*3, which classified them into class 1. Despite the variation in the arrangement of the *Cas* protein and the absence of *cas*4, the two complete systems could further be classified into subtype 1-E (Figure 7A). Approximately 90% (*n* = 18) of the *Paraburkholderia* species had so-called orphan CRISPRs (those not associated with *Cas* genes or remnants of CRISPR systems), containing at least two spacers and three repeats. The genome of *P. oxyphila* had the highest number of spacers, with 36 and 37 repeats (see Table 4 and Supplementary Figure 5).

**Table 4:** Summary of CRISPR elements found across all *Paraburkholderia* genomes in this study.

**Host**	**Strain**	**#CRISPR systems**	**Total #spacers**	**# Repeat sequences**	**Repeat length (bp)**	**Repeat number**	**Repeat Sequence**	**Cas genes^a^**
*P. terrae*	BS001	2	5	2	23	1	CGCGGATGCCAGCGCAAAGGCAA	NI
					25	2	GCGTAAGCGCTAAAGCGCTAACGCC	NI
*P. terrae*	BS007	2	5	2	25	3	GGCGTTAGCGCTTTAGTGCTTACGC	NI
					23	4	CATAACGCGGATGCCAGCGCAAA	NI
*P. terrae*	BS110	2	5	2	23	5	CGCGGATGCCAGCGCAAAGGCAA	NI
					25	6	GCGTAAGCGCTAAAGCGCTAACGCC	NI
*P. terrae*	DSM 17804^T^	2	2	2	24	7	GTTTGCGCTGGCATCCGCGATTTG	NI
					23	8	TGCACAAACAACCTCACCTTCCT1	NI
*P. terrae*	NBRC 100964	2	5	2	24	27	GTTTGCGCTGGCATCCGCGATTTG	NI
					23	28	TGCACAAACAACCTCACCTTCCT	NI
*P. graminis*	C4D1M	1	2	1	24	9	GAACCCGCAGAACCCGCAGAACCC	NI
*P. grimmiae*	R27	2	42	2	29	10	GGGTCTATCTCCGCGCACGCGGAGGAACC	*cas*1, *cas*2, *cas*3HR,
					29	11	GGTTCCTCCGCATGCGCGGAGATAGACCC	*cas*5, *cas*6e, *cas*7, *ca*s8e, and cse2gr11
*P. heleia*	NBRC 101817	1	3	1	24	12	TACCACGGCGGCTACTATCATGGC	NI
*P. nodosa*	DSM 21604	1	2	1	32	13	TGCTCGTGCTCGTGCTCGTGCTCGTGCTCGTG	NI
*P. phymatum*	stm815	1	2	1	23	14	GGCGGCAACCGCGAAGGCGGCTA	NI
*P. phytofirmans*	PsJN	1	3	1	23	15	TTCGTACCCGATCGGGTACGAAA	NI
*P. phytofirmans*	J1U5	1	7	1	24	16	AGTCCGGTGACCGGCGCGAGCGGA	NI
*P. sacchari*	LMG 19450	2	8	2	24	17^*^	GAAAAGTGACGGATTGTGGCCCGC	NI
					24	18^*^	GAAAAGTGACGGATTGTGGCCCGC	NI
*P. sprentiae*	WSM5005	1	2	1	24	19	GGCTAAACCGAGCGCCATACTTGC	NI
*P. hospita*	DSM 17164^T^	1	3	1	25	20	GGCGTTAGCGCTTTAGTGCTTACGC	NI
*P. oxyphila*	NBRC 105797	3	36	3	28	21	TGTGTCGACTCGACACAGCACTCAATCG	NI
					28	22	TTTCTAAGCTGCCTACGCGGCAGCGAAC	NI
					24	23	GTCGACCAGAGTTAGCGCTTCAGC	NI
*P. phenoliruptrix*	AC1100	1	2	1	24	24	TTGTCCACGTGTATCCGCTCAAAT	NI
*Paraburkholderia* sp.	MF2-27	2	7	2	27	25	GGTCAGCGGTGCCAGCGGGCTGCTGCC	NI
					24	26	TTGTCCACGTGTATCCGCTCAAAT	NI
*P. xenovorans*	LB400	2	5	2	25	29	CTTCGTACCCGAGCGGGTACGAAAT	NI
					24	30	AAAGGTGAGCGTTTTCGGGAGCGC	NI
*P. zhejiangensis*	CEIB S4-3	2	18	2	28	31	GGTCTATCTCCGCGCGCGCGGAGGAACC	*cas*1, *cas*2, *cas*3HR,
					25	32	GGTTCCTCCGCGTCCGCGGAGATAG	*cas*5, *cas*6e, *cas*7, *ca*s8e, and cse2gr11

a NI,not identified;

* *named on the basis of different location in the genome*.

Given the high percentage of orphan CRISPRs, we then also classified the systems found on the basis of the repeat sequences found in the CRISPR arrays (see Supplementary Figure 5) using the CRISPRmap database (Lange et al., 2013). Based on the analyses, most repeats belonged to super-classes D (46.87%, *n* = 15) and B (12.5%, *n* = 4). Moreover, 37.5% (*n* = 12) of the repeats were not attributable to any superclass. Additionally, none (*n* = 32) of the repeats showed a match with any sequence family and/or structure motif in the CRISPRmap database (Lange et al., 2013). Furthermore, repeat30 from *P. xenovorans* was found to match motif11 in the database (Supplementary Figure 6). In detail, this repeat was related to a repeat found in CRISPR-Cas systems of the Gram-positive bacteria *Streptococcus pneumoniae* CGSP14 (NC_010582) and *Clostridium botulinum* F str. 230613 (NC_017297) (see Supplementary Figure 6 and Supplementary Tables 2, 3). Moreover, the highest numbers of repeats were found in the *P. grimmiae* genome, which harbored 44 repeat copies. These consisted of two consensus repeats, i.e., 12 copies of repeat10: GGGTCTATCTCCGCGCACGCGGAGGAACC and 32 of repeat11: GGTTCCTCCGCATGCGCGGAGATAGACCC. Both repeats had lengths of 29 bp (Table 4). The repeat with next-high number was found in *P. oxyphila*. This genome harbored 39 repeats, consisting of three consensus sequences. These were: five copies of repeat21: TGTGTCGACTCGACACAGCACTCAATCG, 30 of repeat22: TTTCTAAGCTGCCTACGCGGCAGCGAAC and five of repeat23: GTCGACCAGAGTTAGCGCTTCAGC. These repeats had lengths of 28, 28, and 24 bp, respectively (Table 4). The genome of *P. zhejiangensis* also harbored relatively high repeat sequence numbers, with a total of 20 repeat sequences. These consisted of two consensus repeats, i.e., 12 copies of repeat31: GGTCTATCTCCGCGCGCGCGGAGGAACC and eight of repeat32: GGTTCCTCCGCGTCCGCGGAGATAG. These repeats had lengths of 28 and 25 bp, respectively. Remarkably, we found similar CRISPR-array repeat sequences in some of the *Paraburkholderia* genomes, i.e. repeat24 from *P. phenoliruptrix* ac1100 was similar to repeat26 from *Paraburkholderia* sp. MF2-27, as well as repeat1 and repeat2 from *P. terrae* strain BS001 were similar to repeat5 and repeat6 from *P. terrae* strain BS110, respectively.

In order to discern the phages (and other mobile genetic elements) that most frequently infect the genomes of *Paraburkholderia* spp., we compared the spacer matches to phage, virus and plasmid sequences found in the database (NCBI). Based on the numbers of phages from different families (Figure 7B), we found that 31.14% (*n* = 52/167) of the spacers had best hits against database sequences, with 115 spacers remaining unknown. This analysis thus identified sequences of *Coronaviridae, Flaviviridae, Geminiviridae, Herpeviridae, Inoviridae, Myoviridae, Podoviridae*, and *Siphoviridae*. For example, most *Myoviridae* phages came from Gamma-Proteobacterial hosts, such as *Burkholderia, Pseudomonas*, and *Erwinia* (see Figure 7B; the detailed organism hits can be seen in Supplementary Table 4). Spacers with the highest hits often matched phages from the family *Myoviridae* (21%, *n* = 11). The comparison of the spacer dataset to the prophage dataset using BLAST (all-vs.-all) did not yield any matches. In the analyses, we were unable to detect any other mobile genetic elements (Supplementary Table 4).

To investigate whether the identified prophages could be predicted to infect hosts beyond *Paraburkholderia* spp., we compared our prophage dataset against the viral and metagenomics spacer database in the IMG/VR platform (Paez-Espino et al., 2017). Remarkably, most of the identified prophages were found to have a narrow predicted host range, as evidenced by the absence of matches to other bacteria. However, there were some exceptions. For instance, phage ϕ437 was found to contain two proto-spacers with 100% identity to spacers in the genomes of *Yersinia pekkanenii* strains A125KOH2 and CIP110230 (see Figure 7C and Supplementary Table 5). Other phages, i.e., ϕPban1, ϕPban3, ϕPsp21, ϕPsp31, and ϕPsp51, contained proto-spacers with 95–100% matches to spacers present in the metagenomics spacer database, specifically from maize rhizosphere and peatland microbiomes (Supplementary Table 6).

### Screening for R-M defense systems

**Figure 8:**
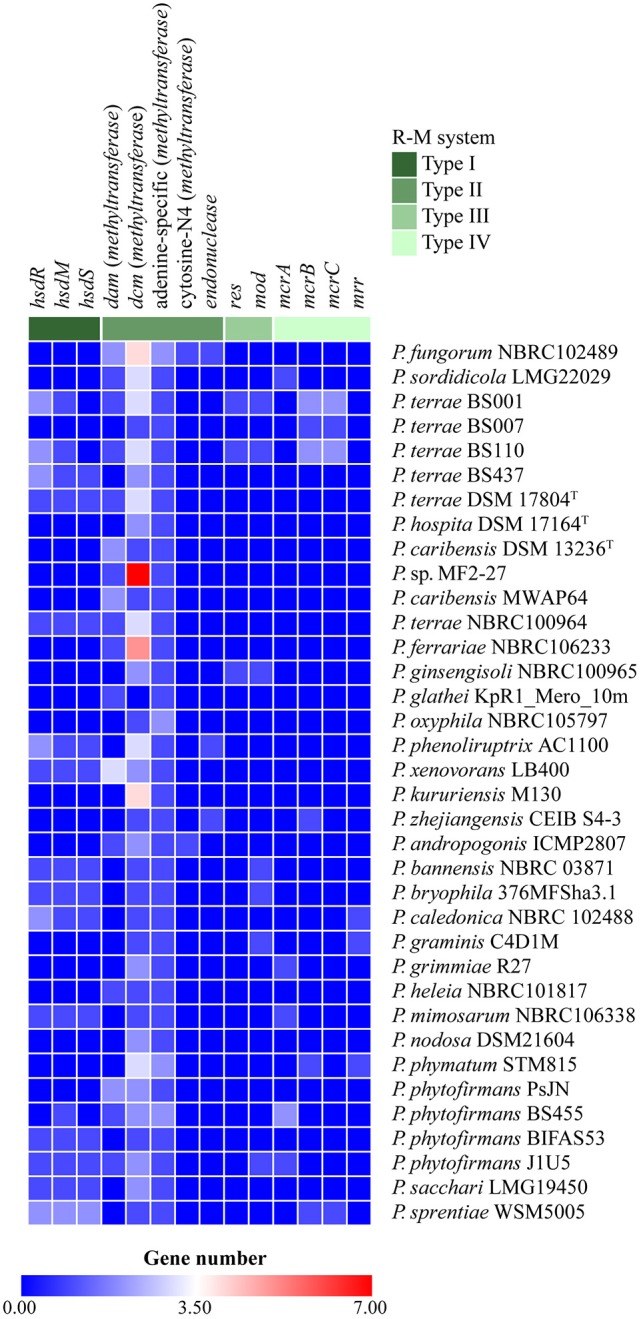
Heat map representing restriction-modification (R-M) systems found in the *Paraburkholderia* species. The rows represent the genomes tested for the presence of genes in R-M systems. The horizontal axis represents gene numbers in R-M systems. The color scale represents the number of genes, with dark blue squares representing the absence of matches and red squares representing the highest numbers of matches.

We found genes involved in R-M systems of types I, II, III, and IV across the *Paraburkholderia* genomes. Our results showed that type-II R-M systems were widely found in all genomes, next to type-I ones (Figure 8). Further, only *P. terrae* BS001, BS007 and *P. phytofirmans* J1U5 contained all R-M system types (I–IV). Additionally, we found *P. terrae* strain BS437 to only have type-I (i.e., *hsdR, hsdM*, and *hsdS*) and type-II R-M systems (i.e. *dcm*-methyltransferase and adenine-specific-methyltransferase; Figure 8).

## Discussion

In this study, we addressed the question to what extent phages have shaped, over evolutionary time, the genomes of *Paraburkholderia* species. It has been amply shown that the genomes of bacteria are often littered with both functional and “fossilized” viral sequences (Casjens, 2003). Here, prophage sequences were indeed found in the majority of the *Paraburkholderia* genomes (Table 1). In most cases, we found evidence for genetic degradation, most likely due to the hosts' selective pressure leading to deletions (deletion bias). Remarkably, in just a few *Paraburkholderia* genomes we found multiple prophage regions, whereas most were found to contain just one such region (see Table 2 and Supplementary Figure 1).

A previous study on *Paraburkholderia* genomes showed that prophages can make up to 13% of these, as exemplified by the *P. phytofirmans* J1U5 genome (Pratama and van Elsas, 2017). It is worth to note that this previous study used not only the database-based approach (i.e., PHAST) but also an algorithm-based program (i.e., PhiSpy) to find novel prophage sequences. However, the latter program has been reported to give less consistent results (Popa et al., 2017; Pratama and van Elsas, 2017). Therefore, in this current study we decided to identify prophages based on more strict criteria, as outlined in section Materials and Methods. Moreover, we decided to base our analyses solely on the latest prophage/viral database (Zhou et al., 2011).

Our current analysis shows that there was no significant difference between the size of the prophage regions found in the genomes of the plant-associated vs. the non-plant-associated *Paraburkholderia* strains (including soil and mycosphere inhabitants; Figure 2). It may support the notion that the phenotypic diversity of *Paraburkholderia* species enables them to inhabit diverse soil environmental settings. In consequence, at some point of their lifetime, they may have been exposed to either different or similar phage pools, allowing the acquisition of diverse novel sequences by phage insertions. The latter may thus relate to the lifestyles of these organisms in soil. Examples can be observed in *Paraburkholderia* sp. MF2-27 that was isolated from the mycosphere of *Trichoderma harzianum* (Rudnick et al., 2015) and was found to contain the highest prophage number in our dataset. This *Paraburkholderia* genome harbors nine prophage sequences, six of these being complete prophages. Clearly, its phage exposure legacy was different from that of the other hosts that were examined, which potentially reflects a more “turbulent” evolutionary record. In contrast, the plant-associated *Paraburkholderia* species containing the highest number of prophages was *P. phymatum* STM815, with a total of five prophages, two of which were complete (see Tables 1, 2).

Furthermore, the G+C contents of all full prophage regions were lower than those of the genomes of their host. This was taken to reflect their relatively “recent” acquisition on the evolutionary time scale (Hendrix et al., 2001; Casjens, 2003; Canchaya et al., 2004). It is worth to mention that - to the best of our knowledge—there is still a lack of reliable estimation of the time scale between phage integration and codon usage equilibrium in the host genomes. Moreover, some of the phages—within their taxon—were highly syntenous across each other (e.g., ϕPt17804 and ϕPtNBRC1; ϕPphytPsJN and ϕPhyt455), suggesting that these were (i) preserved, possibly functionally, and (ii) vertically inherited. These prophages may have derived from a single ancestral integration and then maintained through different diversification events (Bobay et al., 2013). The overall results of prophage distributions and prophage genome architectures suggested that possibly multiple infections by distinct prophages of the respective host cells had taken place. In an overall fashion, the genetic history of these *Paraburkholderia* prophages was found to be very complex, as was previously also observed in the genetic history of *E. coli* prophages (Ohnishi et al., 2001).

In a recent paper, we described that a novel prophage—denoted ϕ437—could be induced from *P. terrae* strain BS437 (Pratama and van Elsas, 2017). We found that it harbors the putative moron gene *amrZ* and we hypothesized that this gene enhances the host's biofilm formation capacity. In the current study, we also found moron genes in other prophage genomes (see Supplementary Table 1). For example, genes for methylases were found and such proteins may be important for phages to overcome bacterial R-M systems, maintain the phage lysogenic stage, as well as support host pathogenicity (Murphy et al., 2013). Experimental studies are required to prove this.

Interestingly, the co-phylogenetic analyses (global-fit) between *Paraburkholderia* and their prophages revealed incongruence between trees (see Table 3). This means that the evolutionary events shaping the phage-host partnerships may have been duplications, host-switching and horizontal gene transfer (HGT) events. Jane results showed co-speciation, duplication, host switching and phage losses had differentially occurred (see Figure 6 and Table 3). Both analyses, thus, indicated that all host-prophage links below the set threshold (Figure 5B) were corresponding to co-speciation (Figure 6; exceptions being ϕPphym1 and ϕPphym1). There are several scenarios under which host switching can occur in the natural environment. For example, ϕ437 may have switched from *Paraburkholderia* sp. MF2-27 to *P. terrae* strain BS437 (Figure 6). The two organisms were, interestingly, isolated from the mycosphere. Moreover, five host switch events were suggested to have occurred from “plant-associated” to non-plant-associated *Paraburkholderia* species, while three (plant to plant) and two (non-plant to non-plant) were observed from *Paraburkholderia* living in the same habitat (see section Materials and Methods for “plant-” vs. “non-plant-associated” *Paraburkholderia* species). These results support the notion of the ecological plasticity of *Paraburkholderia* species to occupy different niches in the soil (Haq et al., 2014). We also hypothesize that these *Paraburkholderia* (*P. terrae* strain BS437 and *Paraburkholderia* sp. MF2-27) might have been in close proximity and exposed to diverse prophages. The latter may thus have infected, and diverged in, these *Paraburkholderia* species, either including or not including transfers between species. Different rates of phage evolution could have been caused by (i) the variations in host evolution itself, (ii) accessibility of the common (horizontal) gene pool in different environments, (iii) constraints on the sequence diversity present across the genomes and available for recombination and (iv) the roles of temperate phages (i.e., high in gene flux—faster rate of gene acquisition and loss through HGT; Mavrich and Hatfull, 2017).

Our CRISPR-Cas analyses showed the infestation record of past infections by exogenous DNA elements (Figure 7). Strikingly, we found high variability of the CRISPR systems, with an uneven presence and numbers of CRISPR spacers. We surmised this is the consequence of differential *Paraburkholderia* arms races with exogenous elements like phages (see Table 4 and Figure 7). It is speculated that high variability of CRISP-Cas systems is due to (i) a high rate of HGT in some of these species “hubs,” (ii) possible duplication of the arrays and (iii) the enrichment of these arrays offering other advantages to the host cell (Weissman et al., 2017). Interestingly, *Paraburkholderia* species with complete CRISPR-Cas systems, i.e., *P. grimmiae* and *P. zhejiangensis*, grouped in the same branch (Figure 1), suggesting their close relationship. We also observed the composition of the genes for *Cas* proteins in *P. grimmiae* and *P. zhejiangensis* to be different from the classification proposed by Makarova et al. (2015).

The finding of high numbers of orphan CRISPRs in the analyzed genomes was remarkable. It could be the consequence of (i) rapid genetic rearrangement in the bacterial genome, (ii) the loss of functionality of CRISPR sequences by deletion,(iii) an incomplete or poor assembly of the draft genome (Shin et al., 2017) and (iv) lifestyle of the bacteria (Makarova et al., 2015). To date, only ~40% of bacterial genome sequences in the current database were found to carry CRISPR systems. Moreover, it is still unknown why the genetic structures of CRISPR-Cas systems are so diverse and have such a non-uniform distribution (Vale and Little, 2010; Makarova et al., 2015). The finding of CRISPR arrays in only half of our *Paraburkholderia* genomes (see Table 4 and Figure 7), was thus not unexpected, as we may be just lifting the tip of the iceberg of *Paraburkholderia* CRISPR array sequences.

Furthermore, we clearly found evidence for the tenet that the host CRISPR spacers matched sequences of a variety of phage families (Figure 7B). Matches to *Myoviridae* were the highest, suggesting phages from this family most commonly infected the *Paraburkholderia* species. This result was consistent with the recent discovery of the inducible *Myoviridae* phage ϕ437 from *P. terrae* BS437 (Pratama and van Elsas, 2017). The matches with various different viruses and phages (see Supplementary Table 1 for details of the hosts of these viruses and phages) showed some were hits with known *Burkholderia* phages (e.g., *Burkholderia cepacia* complex phages BcepC6B and phiE12-2) and other Proteobacteria phages (e.g., *Pseudomonas* phage Lu11 and PEV2). These results confirm the contention that most of the CRISPR spacers have been exposed to rapid genetic turnover processes (Makarova et al., 2015; Shmakov et al., 2017). We argue here that, given that phages indeed constituted the majority of matches of the spacers, they most likely constitute the main mobile genetic elements the *Paraburkholderia* hosts have been exposed to, as argued in Modell et al. (2017), Shin et al. (2017) and Shmakov et al. (2017). However, we acknowledge the paucity of knowledge on spacers in databases, as well as the limitations posed by current bioinformatics analysis programs. And, although we have come a long way in our understanding of the CRISPR-Cas systems of prokaryotes (bacteria and archaea), these still remain to be better explored and identified.

The clear matches of the proto-spacers in phage ϕ437 with genome sequences of *Y. pekkanenii* and with other phages in the metagenomics database suggest an co-evolutionary relationship. Either such phages may have a surprisingly broad host range, or the two divergent organisms may have been infected with related phages in their natural ecosystem. *Y. pekkanenii* has been isolated from soil (Murros-Kontiainen et al., 2011), suggesting a common niche. Niche sharing by *P. terrae* BS437 and *Y. pekkanenii* may, thus, be at the basis of the relatedness. However, these tenets are speculative and need confirmation.

The fact that many *Paraburkholderia* strains (i.e., *P. grimmiae, P. zhejiangenis, P. terrae* BS001, *P. terrae* BS007, *P. terrae* BS110, *P. terrae* DSM 17804^T^, *P. terrae* NBRC 100964, *P. xenovorans, Paraburkholderia* sp. MF2-27, *P. sacchari*, and *P. oxyphila*) had multiple CRISPR arrays (Figure 7A) indicated multiple exposures to phages. Moreover, *Paraburkholderia* may also have utilized other antiphage defense systems, such as the R-M system (Figure 8). Recent studies have reported previously unknown anti-phage systems, next to one anti-plasmid system, that are widespread and arm bacterial genomes against invading genetic elements like phages and plasmids (Doron et al., 2018; Ofir et al., 2018). We currently ignore the extent to which such systems are operational in *Paraburkholderia* species and so further analyses on these systems are warranted.

In summary, we here analyzed the distribution of prophage regions across the genomes of all species of the genus *Paraburkholderia*. Although we observed incongruences between the trees built for host and prophage evolutionary relationships, we obtained evidence for the tenet that duplication, host switching and HGT have affected the evolutionary histories. The analyses of CRISPR-Cas systems also indicated frequent phage-host encounters, revealing a complex and entangled relationship.

## Author contributions

AP and JE: Conceived the study and supervised manuscript preparation; AP: Performed the analyses; MC: Supported the analyses; AP: Prepared all tables and figures; AP, MC, and JE: Interpreted the data and drafted the manuscript.

### Conflict of interest statement

The authors declare that the research was conducted in the absence of any commercial or financial relationships that could be construed as a potential conflict of interest.

## References

[B1] Almendros C. Guzmán N. M. García-Martínez J. Mojica F. J. (2016). Anti-cas spacers in orphan CRISPR4 arrays prevent uptake of active CRISPR–Cas I-F systems. Nat. Microbiol. 1:16081. 10.1038/nmicrobiol.2016.81 27573106

[B2] Altschul S. F. Madden T. L. Schäffer A. A. Zhang J. Zhang Z. Miller W.. (1997). Gapped BLAST and PSI-BLAST: a new generation of protein database search programs. Nucleic Acids Res. 25, 3389–3402. 10.1093/nar/25.17.3389 9254694 PMC146917

[B3] Balbuena J. A. Míguez-Lozano R. Blasco-Costa I. (2013). PACo: a novel procrustes application to cophylogenetic analysis. PLoS ONE 8:e61048. 10.1371/journal.pone.0061048 23580325 PMC3620278

[B4] Bobay L. M. Rocha E. P. Touchon M. (2013). The adaptation of temperate bacteriophages to their host genomes. Mol. Biol. Evol. 30, 737–751. 10.1093/molbev/mss279 23243039 PMC3603311

[B5] Bobay L.-M. Touchon M. Rocha E. P. (2014). Pervasive domestication of defective prophages by bacteria. Proc. Natl. Acad. Sci. U.S.A. 111, 12127–12132. 10.1073/pnas.1405336111 25092302 PMC4143005

[B6] Brüssow H. Canchaya C. Hardt W. Bru H. (2004). Phages and the evolution of bacterial pathogens : from genomic rearrangements to lysogenic conversion. Microbiol. Mol. Biol. Rev. 68, 560–602. 10.1128/MMBR.68.3.560-602.2004 15353570 PMC515249

[B7] Canchaya C. Fournous G. Brüssow H. (2004). The impact of prophages on bacterial chromosomes. Mol. Microbiol. 53, 9–18. 10.1111/j.1365-2958.2004.04113.x 15225299

[B8] Casjens S. (2003). Prophages and bacterial genomics: what have we learned so far? Mol. Microbiol. 49, 277–300. 10.1046/j.1365-2958.2003.03580.x 12886937

[B9] Conow C. Fielder D. Ovadia Y. Libeskind-Hadas R. (2010). Jane: a new tool for the cophylogeny reconstruction problem. Algorithms Mol. Biol. 5:16. 10.1186/1748-7188-5-16 20181081 PMC2830923

[B10] Doron S. Melamed S. Ofir G. Leavitt A. Lopatine A. Keren M.. (2018). Systematic discovery of antiphage defense systems in the microbial pangenome. Science 359:4120. 10.1126/science.aar4120 29371424 PMC6387622

[B11] Edgar R. C. (2004). MUSCLE: multiple sequence alignment with high accuracy and high throughput. Nucleic Acids Res. 32, 1792–1797. 10.1093/nar/gkh340 15034147 PMC390337

[B12] Edwards R. A. McNair K. Faust K. Raes J. Dutilh B. E. (2016). Computational approaches to predict bacteriophage-host relationships. FEMS Microbiol. Rev. 40, 258–272. 10.1093/femsre/fuv048 26657537 PMC5831537

[B13] Geoghegan J. L. Duchêne S. Holmes E. C. (2017). Comparative analysis estimates the relative frequencies of co-divergence and cross-species transmission within viral families. PLoS Pathog. 13:e1006215. 10.1371/journal.ppat.1006215 28178344 PMC5319820

[B14] Grissa I. Vergnaud G. Pourcel C. (2008). CRISPRFinder: a website to compare clustered regularly interspaced short palindromic repeats. Nucleic Acids Res. 36, 52–57. 10.1093/nar/gkn228 PMC2447796 18442988

[B15] Haq I. U. Graupner K. Nazir R. Van Elsas J. D. (2014). The genome of the fungal-interactive soil bacterium *Burkholderia terrae* BS001- A plethora of outstanding interactive capabilities unveiled. Genome Biol. Evol. 6, 1652–1668. 10.1093/gbe/evu126 24923325 PMC4122924

[B16] Hendrix R. W. Lawrence J. G. Hatfull G. F. Casjens S. (2001). The origins and evolution of viruses. Trends Microbiol. 9:61 10.1016/S0966-842X(00)01934-X 11121760

[B17] Hutchinson M. C. Cagua E. F. Balbuena J. A. Stouffer D. B. Poisot T. (2017). PACo: implementing procrustean approach to cophylogeny in R. Methods Ecol. Evol. 8, 932–940. 10.1111/2041-210X.12736

[B18] Katoh K. Misawa K. Kuma K. Miyata T. (2002). MAFFT: a novel method for rapid multiple sequence alignment based on fast Fourier transform. Nucleic Acids Res. 30, 3059–3066. 10.1093/nar/gkf436 12136088 PMC135756

[B19] Keen E. C. Bliskovsky V. V. Malagon F. Baker J. D. Prince J. S. Klaus J. S.. (2017). Novel “superspreader” bacteriophages promote horizontal gene transfer by transformation. mBio 8, 1–12. 28096488 10.1128/mBio.02115-16 PMC5241400

[B20] Lange S. J. Alkhnbashi O. S. Rose D. Will S. Backofen R. (2013). CRISPRmap: an automated classification of repeat conservation in prokaryotic adaptive immune systems. Nucleic Acids Res. 41, 8034–8044. 10.1093/nar/gkt606 23863837 PMC3783184

[B21] Lawrence J. G. Ochman H. (1998). Molecular archaeology of the *Escherichia coli* genome. Proc. Natl. Acad. Sci. U.S.A. 95, 9413–9417. 10.1073/pnas.95.16.9413 9689094 PMC21352

[B22] Letunic I. Bork P. (2016). Interactive tree of life (iTOL) v3: an online tool for the display and annotation of phylogenetic and other trees. Nucleic Acids Res. 44, W242–W245. 10.1093/nar/gkw290 27095192 PMC4987883

[B23] Makarova K. S. Wolf Y. I. Alkhnbashi O. S. Costa F. Shah S. A. Saunders S. J.. (2015). An updated evolutionary classification of CRISPR–Cas systems. Nat. Rev. Microbiol. 13, 722–736. 10.1038/nrmicro3569 26411297 PMC5426118

[B24] Manrique P. Bolduc B. Walk S. T. van der Oost J. de Vos W. M. Young M. J. (2016). Healthy human gut phageome. Proc. Natl. Acad. Sci. U.S.A. 113, 10400–10405. 10.1073/pnas.1601060113 27573828 PMC5027468

[B25] Mavrich T. N. Hatfull G. F. (2017). Bacteriophage evolution differs by host, lifestyle and genome. Nat. Microbiol. 2:17112. 10.1038/nmicrobiol.2017.112 28692019 PMC5540316

[B26] Modell J. W. Jiang W. Marraffini L. A. (2017). CRISPR–Cas systems exploit viral DNA injection to establish and maintain adaptive immunity. Nature 544, 101–104. 10.1038/nature21719 28355179 PMC5540373

[B27] Murphy J. Mahony J. Ainsworth S. Nauta A. van Sinderen D. (2013). Bacteriophage orphan DNA methyltransferases: insights from their bacterial origin, function, and occurrence. Appl. Environ. Microbiol. 79, 7547–7555. 10.1128/AEM.02229-13 24123737 PMC3837797

[B28] Murros-Kontiainen A. Johansson P. Niskanen T. Fredriksson-Ahomaa M. Korkeala H. Björkroth J. (2011). *Yersinia pekkanenii* sp. nov. Int. J. Syst. Evol. Microbiol. 61, 2363–2367. 10.1099/ijs.0.019984-0 21037033

[B29] Nazir R. Zhang M. de Boer W. van Elsas J. D. (2012). The capacity to comigrate with *Lyophyllum* sp. strain Karsten through different soils is spread among several phylogenetic groups within the genus *Burkholderia*. Soil Biol. Biochem. 50, 221–233. 10.1016/j.soilbio.2012.03.015

[B30] Obeng N. Pratama A. A. Elsas J. D. van (2016). The significance of mutualistic phages for bacterial ecology and evolution. Trends Microbiol. 24, 440–449. 10.1016/j.tim.2015.12.009 26826796

[B31] Ofir G. Melamed S. Sberro H. Mukamel Z. Silverman S. Yaakov G.. (2018). DISARM is a widespread bacterial defence system with broad anti-phage activities. Nat. Microbiol. 3, 90–98. 10.1038/s41564-017-0051-0 29085076 PMC5739279

[B32] Ohnishi M. Kurokawa K. Hayashi T. (2001). Diversification of *Escherichia coli* genomes: are bacteriophages the major contributors? Trends Microbiol. 9, 481–485. 10.1016/S0966-842X(01)02173-4 11597449

[B33] Paez-Espino D. Chen I. A. Palaniappan K. Ratner A. Chu K. Szeto E.. (2017). IMG/VR: a database of cultured and uncultured DNA viruses and retroviruses. Nucleic Acids Res. 45, D457–D465. 10.1093/nar/gkw1030 27799466 PMC5210529

[B34] Pedulla M. L. Ford M. E. Houtz J. M. Karthikeyan T. Wadsworth C. Lewis J. A.. (2003). Origins of highly mosaic mycobacteriophage genomes. Cell 113, 171–182. 10.1016/S0092-8674(03)00233-2 12705866

[B35] Popa O. Landan G. Dagan T. (2017). Phylogenomic networks reveal limited phylogenetic range of lateral gene transfer by transduction. ISME J. 11, 543–554. 10.1038/ismej.2016.116 27648812 PMC5183456

[B36] Pratama A. A. Haq I. U. Nazir R. Chaib De Mares M. van Elsas J. D. (2017). Draft genome sequences of three fungal-interactive *Paraburkholderia* terrae strains, BS007, BS110 and BS437. Stand. Genomic Sci. 12:81. 10.1186/s40793-017-0293-8 29270249 PMC5735546

[B37] Pratama A. A. van Elsas J. D. (2017). A novel inducible prophage from the mycosphere inhabitant *Paraburkholderia terrae* BS437. Sci. Rep. 7:9156. 10.1038/s41598-017-09317-8 28831124 PMC5567305

[B38] Price M. N. Dehal P. S. Arkin A. P. (2009). Fasttree: computing large minimum evolution trees with profiles instead of a distance matrix. Mol. Biol. Evol. 26, 1641–1650. 10.1093/molbev/msp077 19377059 PMC2693737

[B39] Pruesse E. Quast C. Knittel K. Fuchs B. M. Ludwig W. Peplies J.. (2007). SILVA: a comprehensive online resource for quality checked and aligned ribosomal RNA sequence data compatible with ARB. Nucleic Acids Res. 35, 7188–7196. 10.1093/nar/gkm864 17947321 PMC2175337

[B40] Razali N. M. Wah Y. B. (2011). Power comparisons of Shapiro-wilk, kolmogorov-smirnov, lilliefors and Anderson-darling tests. J. Stat. Model. Anal. 2, 21–33.

[B41] Roux S. Brum J. R. Dutilh B. E. Sunagawa S. Duhaime M. B. Loy A.. (2016). Ecogenomics and potential biogeochemical impacts of globally abundant ocean viruses. Nature 537, 689–693. 10.1038/nature19366 27654921

[B42] Rudnick M. B. van Veen J. A. de Boer W. (2015). Baiting of rhizosphere bacteria with hyphae of common soil fungi reveals a diverse group of potentially mycophagous secondary consumers. Soil Biol. Biochem. 88, 73–82. 10.1016/j.soilbio.2015.04.015

[B43] Salmond G. P. Fineran P. C. (2015). A century of the phage: past, present and future. Nat. Rev. Microbiol. 13, 777–786. 10.1038/nrmicro3564 26548913

[B44] Sawana A. Adeolu M. Gupta R. S. (2014). Molecular signatures and phylogenomic analysis of the genus *Burkholderia*: proposal for division of this genus into the emended genus *Burkholderia* containing pathogenic organisms and a new genus *Paraburkholderia* gen. nov. harboring environmental species. Front. Genet. 5:429. 10.3389/fgene.2014.00429 25566316 PMC4271702

[B45] Shin J.-H. Papadimitriou K. Lee J. Barrangou R. Hidalgo-Cantabrana C. Crawley A. B. (2017). Characterization and exploitation of CRISPR loci in *Bifidobacterium longum*. Front. Microbiol. 8:1851 10.3389/fmicb.2017.01851 29033911 PMC5626976

[B46] Shmakov S. A. Sitnik V. Makarova K. S. Wolf Y. I. Severinov K. V. Koonin E. V. (2017). The CRISPR spacer space is dominated by sequences from species-specific mobilomes. mBio 8, 1–18. 10.1128/mBio.01397-17 28928211 PMC5605939

[B47] Stern A. Sorek R. (2011). The phage-host arms race: shaping the evolution of microbes. Bioessays 33, 43–51. 10.1002/bies.201000071 20979102 PMC3274958

[B48] Sullivan M. J. Petty N. K. Beatson S. A. (2011). Easyfig: a genome comparison visualizer. Bioinformatics 27, 1009–1010. 10.1093/bioinformatics/btr039 21278367 PMC3065679

[B49] Sun C. L. Thomas B. C. Barrangou R. Banfield J. F. (2015). Metagenomic reconstructions of bacterial CRISPR loci constrain population histories. ISME J. 10, 858–870. 10.1038/ismej.2015.162 26394009 PMC4796926

[B50] Talavera G. Castresana J. Kjer K. Page R. Sullivan J. (2007). Improvement of phylogenies after removing divergent and ambiguously aligned blocks from protein sequence alignments. Syst. Biol. 56, 564–577. 10.1080/10635150701472164 17654362

[B51] Touchon M. Bernheim A. Rocha E. P. (2016). Genetic and life-history traits associated with the distribution of prophages in bacteria. ISME J. 10, 2744–2754. 10.1038/ismej.2016.47 27015004 PMC5113838

[B52] Vale P. F. Little T. J. (2010). CRISPR-mediated phage resistance and the ghost of coevolution past. Proc. R. Soc. B Biol. Sci. 277, 2097–2103. 10.1098/rspb.2010.0055 20236977 PMC2880148

[B53] Wang J. Ma J. Cheng Z. Meng X. You L. Wang M.. (2016). A CRISPR evolutionary arms race: structural insights into viral anti-CRISPR/Cas responses. Cell Res. 26, 1165–1168. 10.1038/cr.2016.103 27585537 PMC5113301

[B54] Warmink J. A. Nazir R. Corten B. van Elsas J. D (2011). Hitchhikers on the fungal highway: the helper effect for bacterial migration via fungal hyphae. Soil Biol. Biochem. 43, 760–765. 10.1016/j.soilbio.2010.12.009

[B55] Weissman J. L. Fagan W. F. Johnson P. L. F. (2017). Is having more than one CRISPR array adaptive? bioRxiv 10, 23–44. 10.1101/148544

[B56] Zhang M. Visser S. Pereira e Silva M. C. van Elsas J. D. (2014). IncP-1 and PromA group plasmids are major providers of horizontal gene transfer capacities across bacteria in the mycosphere of different soil fungi. Microb. Ecol. 69, 169–179. 10.1007/s00248-014-0482-6 25149284

[B57] Zhang Q. Ye Y. (2017). Not all predicted CRISPR–Cas systems are equal: isolated cas genes and classes of CRISPR like elements. BMC Bioinformatics 18:92. 10.1186/s12859-017-1512-4 28166719 PMC5294841

[B58] Zhou Y. Liang Y. Lynch K. H. Dennis J. J. Wishart D. S. (2011). PHAST: a fast phage search tool. Nucleic Acids Res. 39, 347–352. 10.1093/nar/gkr485 21672955 PMC3125810

