# Supplementary figures and images for: Evolutionary History of Bacteriophages in the Genus Paraburkholderia
Source: Front Microbiol. 2018 May 11;9:835. doi: 10.3389/fmicb.2018.00835 (PMC5968390; doi:10.3389/fmicb.2018.00835)

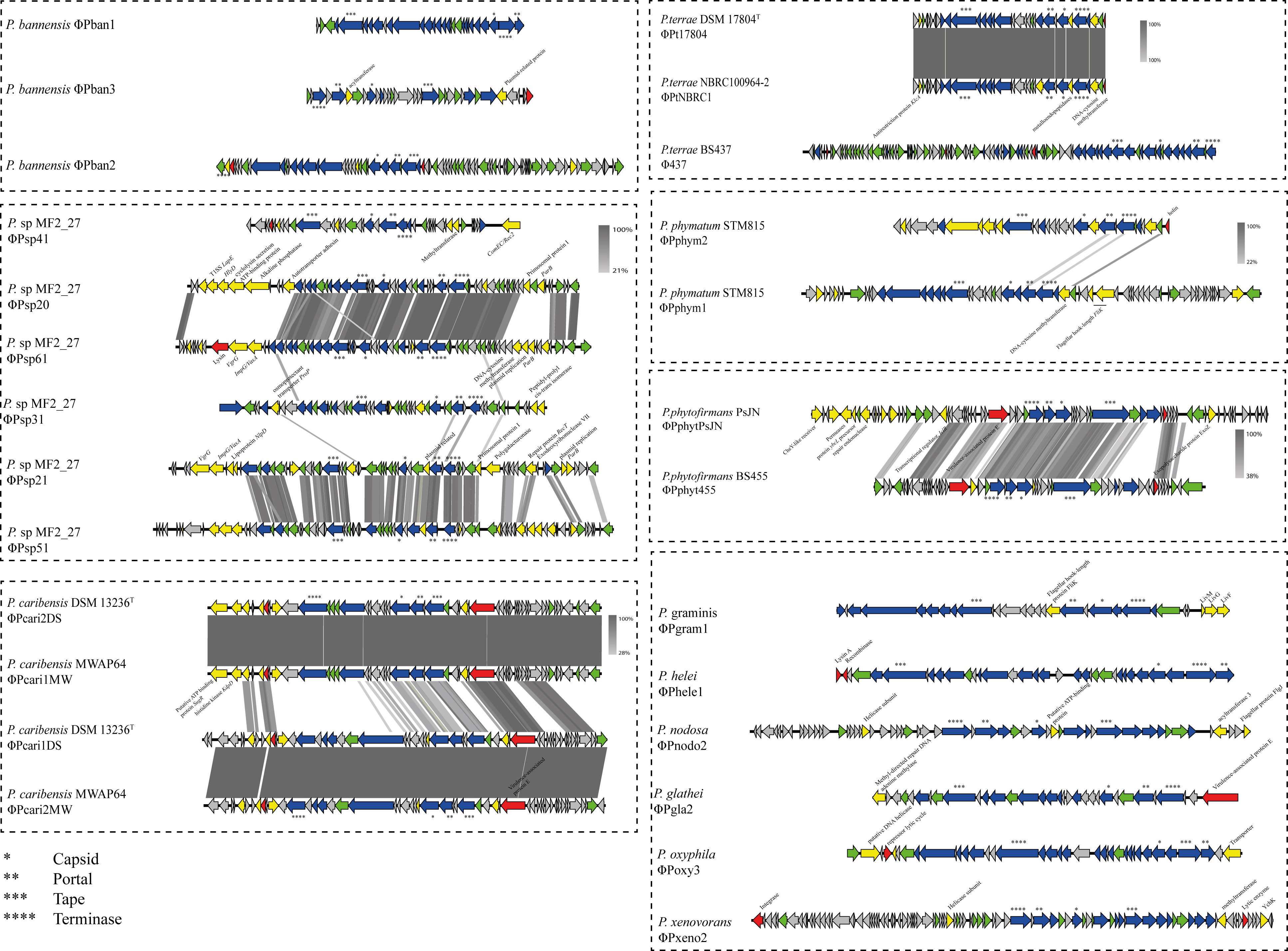

Supplement: Supplementary file 1 [file Image_1.jpg]

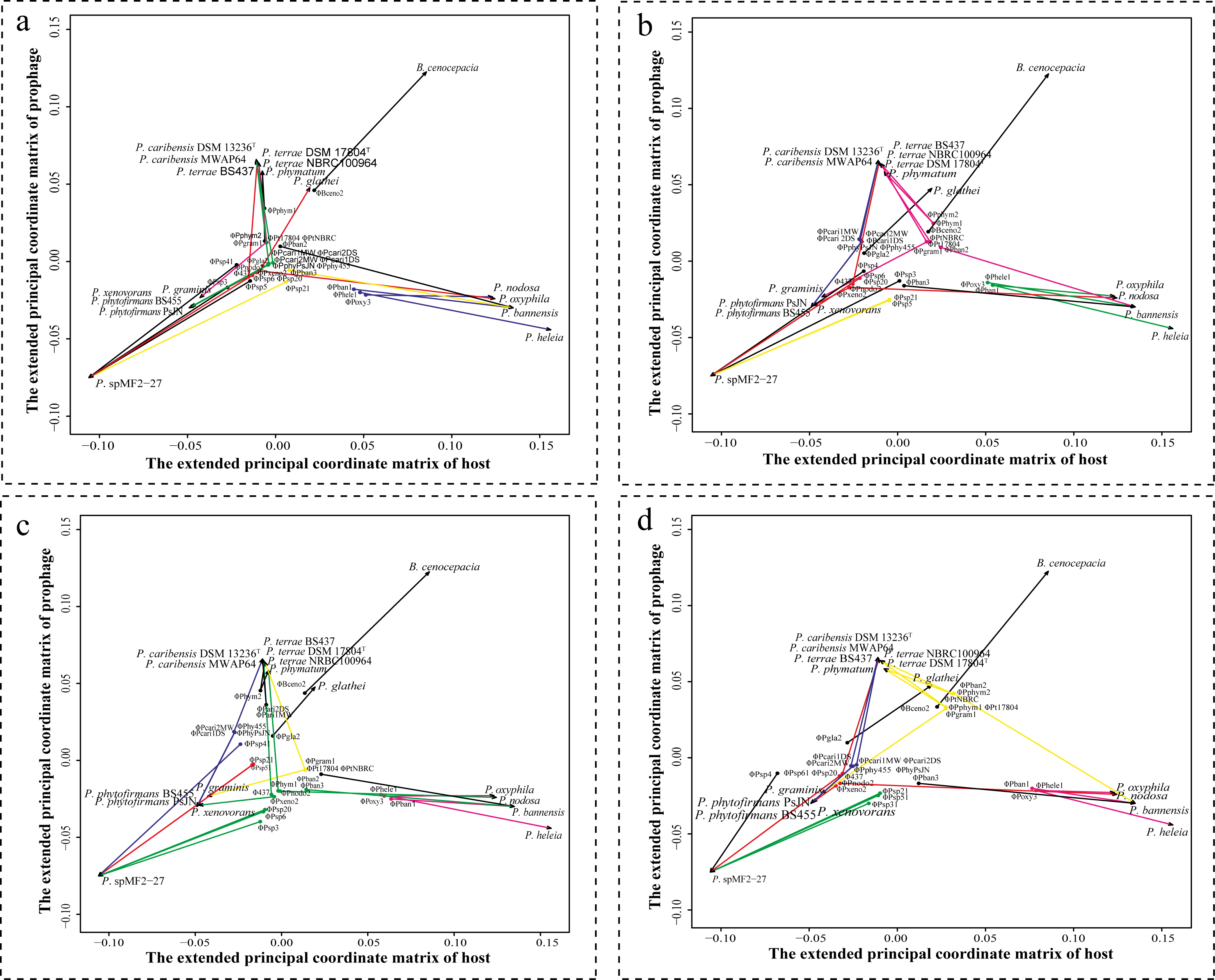

Supplement: Supplementary file 2 [file Image_2.jpg]

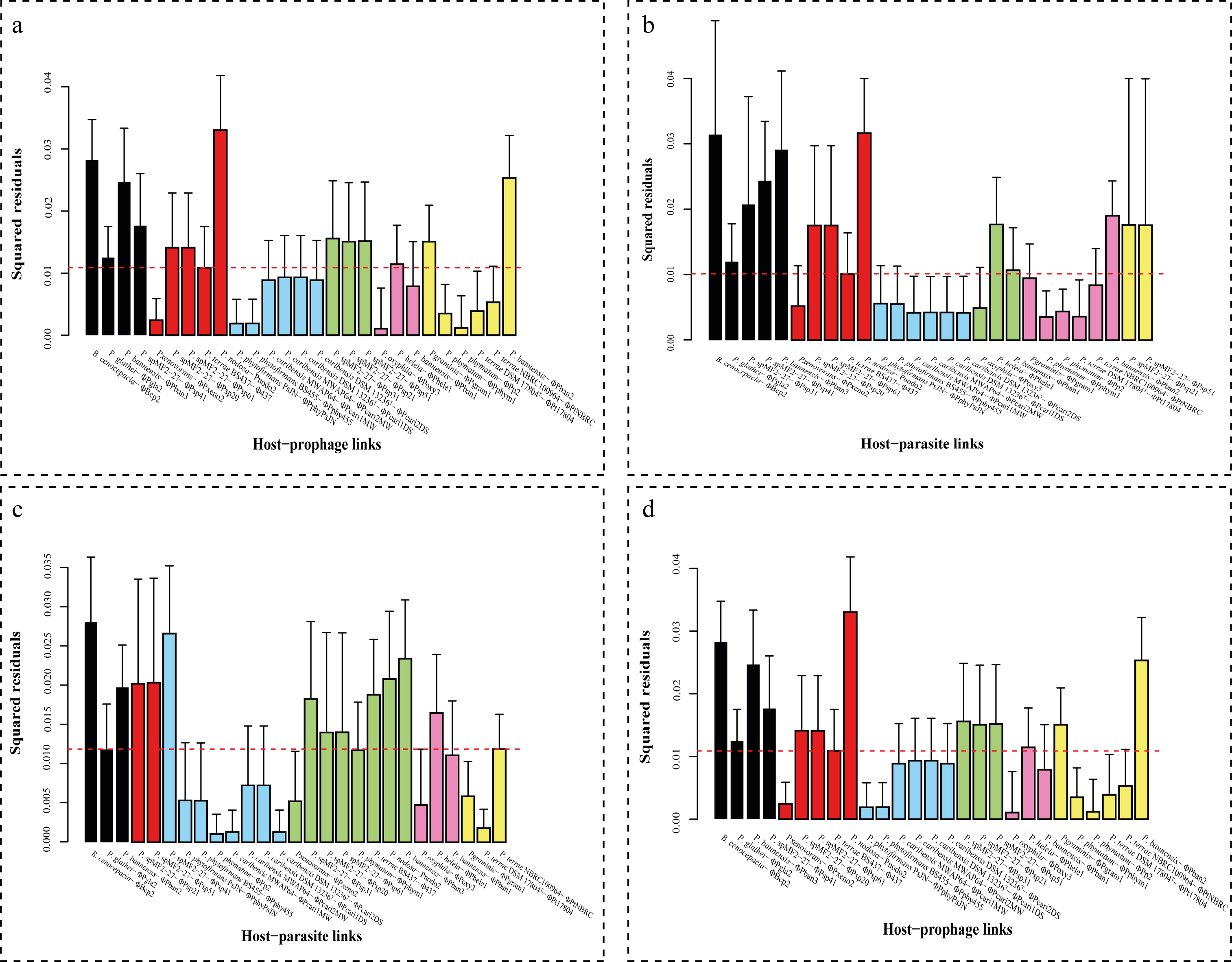

Supplement: Supplementary file 3 [file Image_3.jpg]

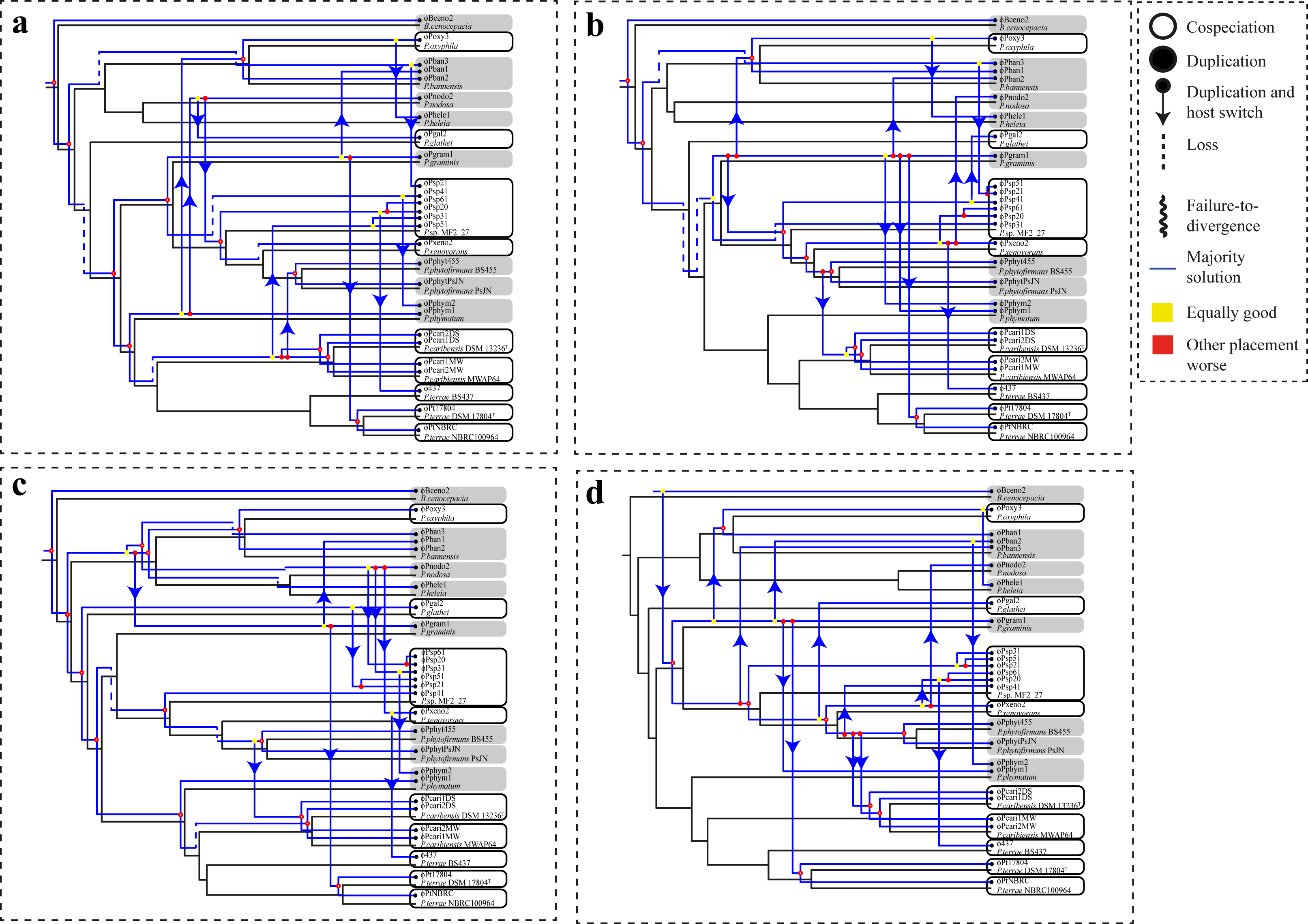

Supplement: Supplementary file 4 [file Image_4.jpg]

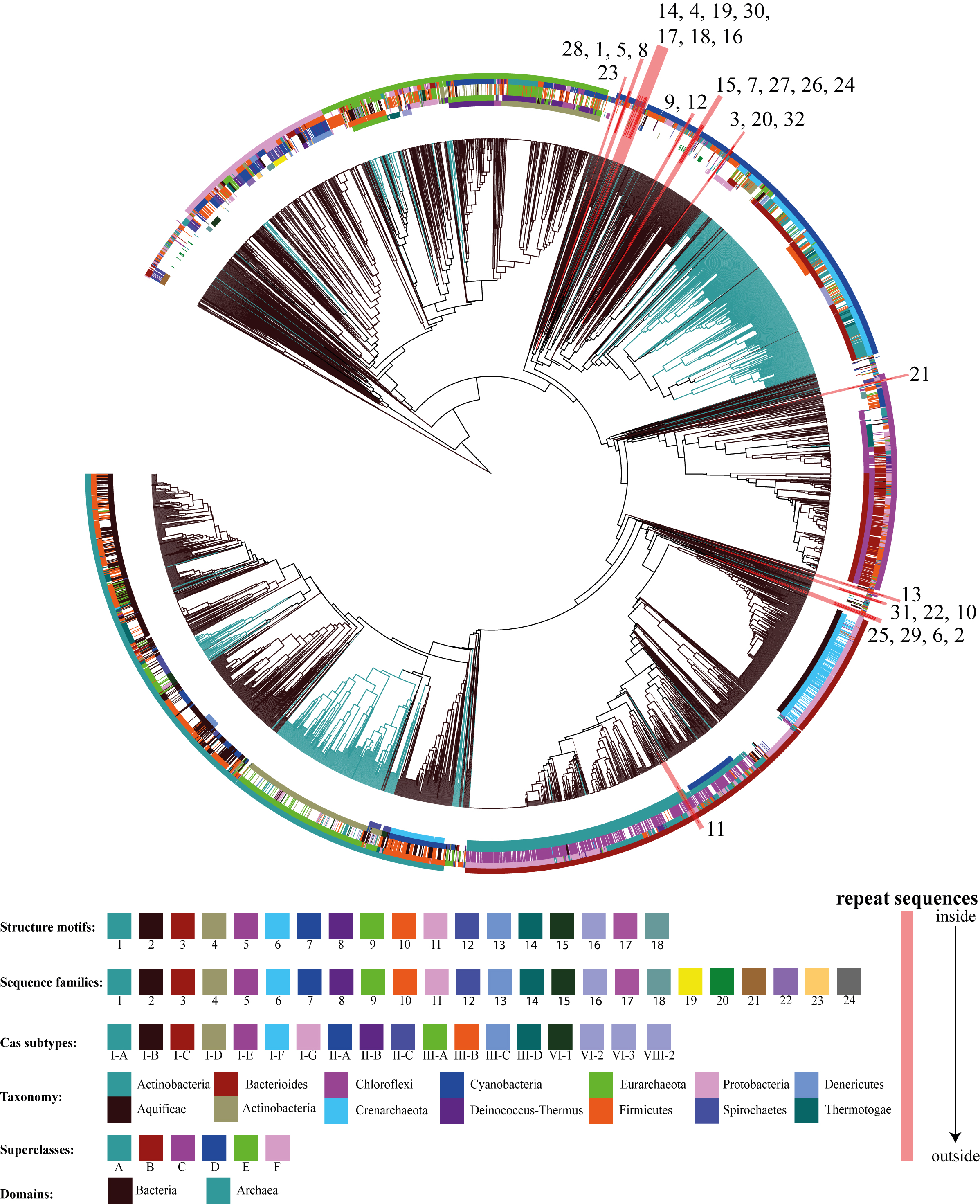

Supplement: Supplementary file 5 [file Image_5.JPEG]

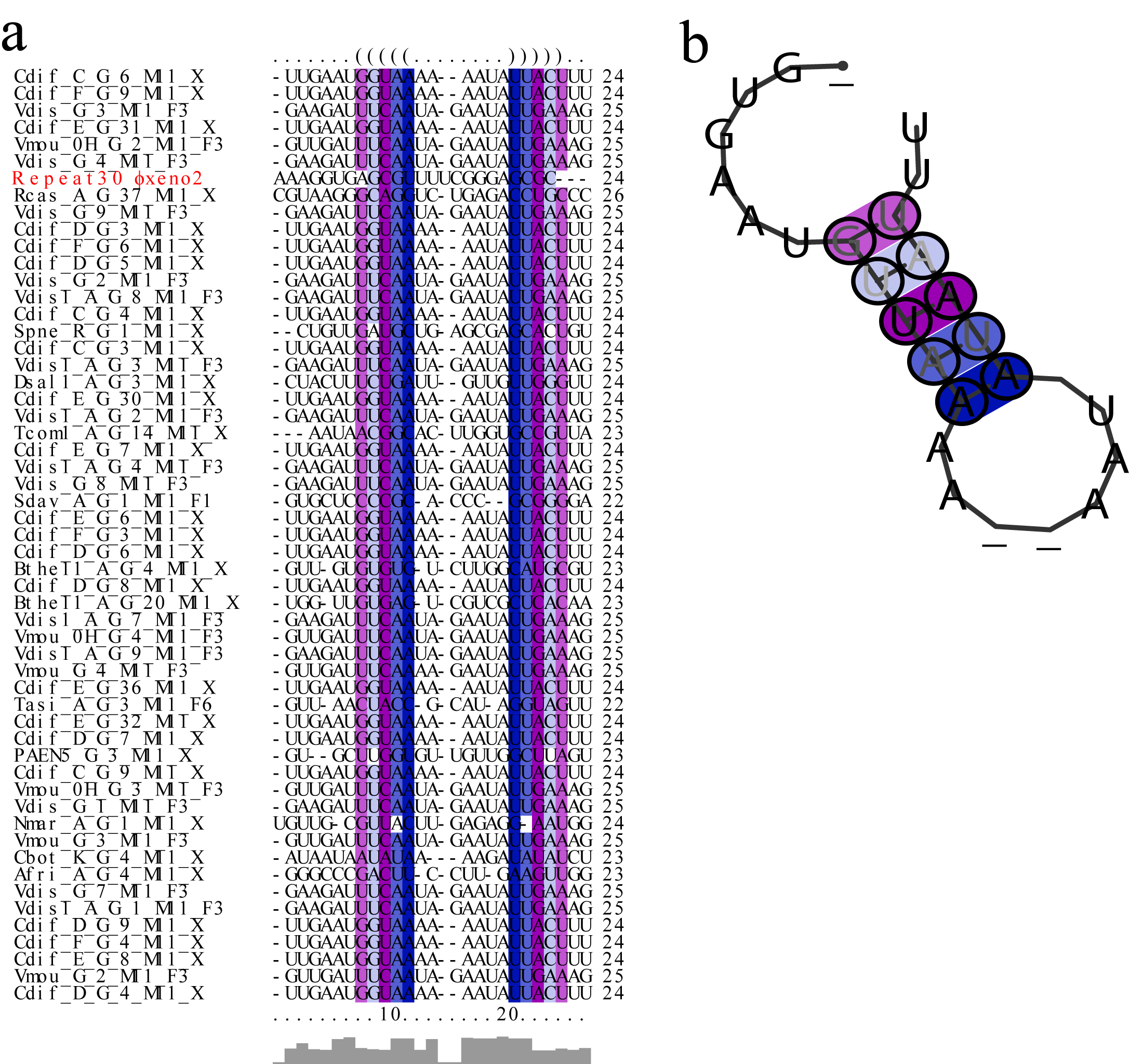

Supplement: Supplementary file 6 [file Image_6.JPEG]
