# Supplementary material for: Evolutionary History of Bacteriophages in the Genus Paraburkholderia
Source: Front Microbiol. 2018 May 11;9:835. doi: 10.3389/fmicb.2018.00835 (PMC5968390; doi:10.3389/fmicb.2018.00835)
Supplement: Supplementary file 8 [file Data_Sheet_1.DOCX]

**SUPPORTING INFORMATION**

**Evolutionary History of Bacteriophages in the Genus *Paraburkholderia***

Akbar Adjie Pratama^1^, Maryam Chaib De Mares^1^, Jan Dirk van Elsas^1^

^1^Department of Microbial Ecology, Microbial Ecology - Groningen Institute for Evolutionary Life Sciences, University of Groningen, Nijenborgh 7, Groningen, AG 9747, The Netherlands

Corresponding authors: AAP (a.a.p.pratama@rug.nl) and JDvE (j.d.van.elsas@rug.nl)

Keywords: *Paraburkholderia*, soil, prophages, mobile genetic elements, evolution

**Supplementary Figure 1**. Synteny analysis of complete prophage genomes per *Paraburkholderia* species. Red arrows: phage lysis and lysogenic genes; blue arrows: phage structural genes (tail, capsid, tail and terminase); green arrows: replication, recombination, repressor and phage related genes; gray arrows: hypothetical proteins and yellow arrows: non-phage genes or possible moron genes. Comparison percentage was generated using BLAST + 2.4.0 (tBLASTx with cutoff value 10−3) and map comparison figures were created with Easyfig (Sullivan et al., 2011) as mentioned in material and methods. Genomes similarity percentage indicated in gray scale bars. The asterisk symbols: (*) capsid, (**) portal, (***) tape and (****) terminase genes.

**Supplementary Figure 2**. PACo procrustean superimposition plot analysis which minimizes differences between the two partners’ principal correspondence coordinates of patristic distances for (a) capsid, (b) portal, (c) tape and (d) terminase genes. For each vector, the starting point (black dot) represents the configuration of prophages and the arrowhead the configuration of hosts. The vector length represents the global fit (residual sum of squares), which is inversely proportional to the topological congruence. The colors represent the consistent groupings shown in the prophage phylogeny (see Figure 3).

**Supplementary Figure 3**. PACo results on the contribution of each *Paraburkholderia* and their prophages to the general pattern of coevolution on (a) capsid, (b) portal, (c) tape and (d) terminase genes. Each bar represents a Jack-knifed squared residual and error bars represent upper 95% confidence intervals from applying PACo to patristic distances. Further, the median squared residual value is shown (dashed red line). The colors represent the clusters shown in the prophage phylogeny (see Fig. 3). Low squared residual values of host-prophage links show dependence of prophages to their hosts, thus suggesting possible co-evolutionary associations.

**Supplementary Figure 4**. Tanglegram depicting the *Paraburkholderia* (host) species phylogenetic trees prophages in black and the prophage tree in blue. Tress based on (a) capsid, (b) portal, (c) tape and (d) terminase gene phylogeny. Auxiliary lines connecting the two trees shown. The event-based method was performed with the default settings for cost regimes (“co-speciation” event = 0; “duplication” event = 1; “loss” event = 1; “duplication then host switching” event = 2) using Jane 4.0 (Conow et al., 2010). All analyses were performed with populations of 1,000 and 10 generations. Grey boxes represent plant-associated *Paraburkholderia* species and white boxes non-plant-associated *Paraburkholderia* species (see materials and methods). Jane results showed that host-switching events occurred frequently next to co-speciation.

**Supplementary Figure 5**. Classification of CRISPR-Cas systems based on the repeat sequences. Circular phylogeny was generated using CRISPRmap (Lange et al., 2013); color rings are explained in the legend below the tree. Bacteria are shown in brown, Archaea in blue-green.

**Supplementary Figure 6**. (a) Multiple sequences alignment (MSA) of the structure of motif11 and (b) The consensus minimum free energy (MFE) structure, of local and global alignment for RNA structure (LocARNA). The analyses were generated using CRISPRmap (Lange et al., 2013).

**Supplementary Table 1**. Potential novel moron genes in prophages identified in genomes of the genus *Paraburkholderia*.

**Supplementary Table 2**. Classification of the CRISPR repeat sequences based on CRISPRmap analysis.

**Supplementary Table 3**. Abbreviation of CRISPR repeat conserved motif11.

**Supplementary Table 4**. CRISPR spacer match hits.

**Supplementary Table 5**. Proto-spacer BLAST analysis against IMG/VR viral spacers database.

**Supplementary Table 6**. Proto-spacer BLAST analysis against IMG/VR metagenomics database.
